# Polyurethane biodegradation by *Fusarium parceramosum* with insights into kinetic modelling and metabolic pathway

**DOI:** 10.1186/s40643-026-01079-4

**Published:** 2026-06-02

**Authors:** Pragya Sahu, Apoorva Sherigar, Ritu Raval, Chuxia Lin, Subbalaxmi Selvaraj

**Affiliations:** 1 Manipal Institute of Technology, Manipal Academy of Higher Education, Manipal, India; 2 Faculty of Science, Engineering, and Built Environment, Deakin University, Burwood, VIC 3125 Australia

**Keywords:** Polyurethane, *Fusarium parceramosum*, Biodegradation, Urease, Aliphatic carbamate hydrolyzing activity, Physicochemical characterization, Kinetic modelling, Metabolic pathway

## Abstract

Polyurethane (PU) is an industrially versatile polymer whose environmental persistence pose significant waste management challenges. Although several PU-degrading microorganisms have been reported, studies evaluating degradation across chemically distinct PU substrate remain limited. The present study explores PU biodegradation potential of a soil-borne fungus *Fusarium parceramosum*, with two chemically different PU substrates—Impranil (PU dispersion), and PU foam—under unoptimized conditions. The fungus achieved 92% degradation of Impranil within 192 h, and 31.29% weight loss of PU foam over 60 d. Degradation kinetics were evaluated using zero-order, first-order, Langmuir, and Freundlich models, with zero-and first-order models showing best fit with experimental data. Enzymatic assay revealed significant urease and aliphatic carbamate-hydrolyzing activities associated with degradation. For Impranil, urease and aliphatic carbamate-hydrolyzing activities were 202.03 U/mL and 412.47 U/mL, respectively, whereas for PU foam they were 338.12 U/mL and 284.17 U/mL, respectively. Structural and chemical alterations were confirmed through SEM, FTIR, XRD and GC–MS analysis. Metabolite identification indicated the formation of degradation intermediates, suggesting their possible involvement in subsequent microbial processes. Overall, this study provides the first evidence of dual-substrate specificity of *Fusarium parceramosum* highlighting its potential in sustainable waste management approaches.

**Graphical abstract:**

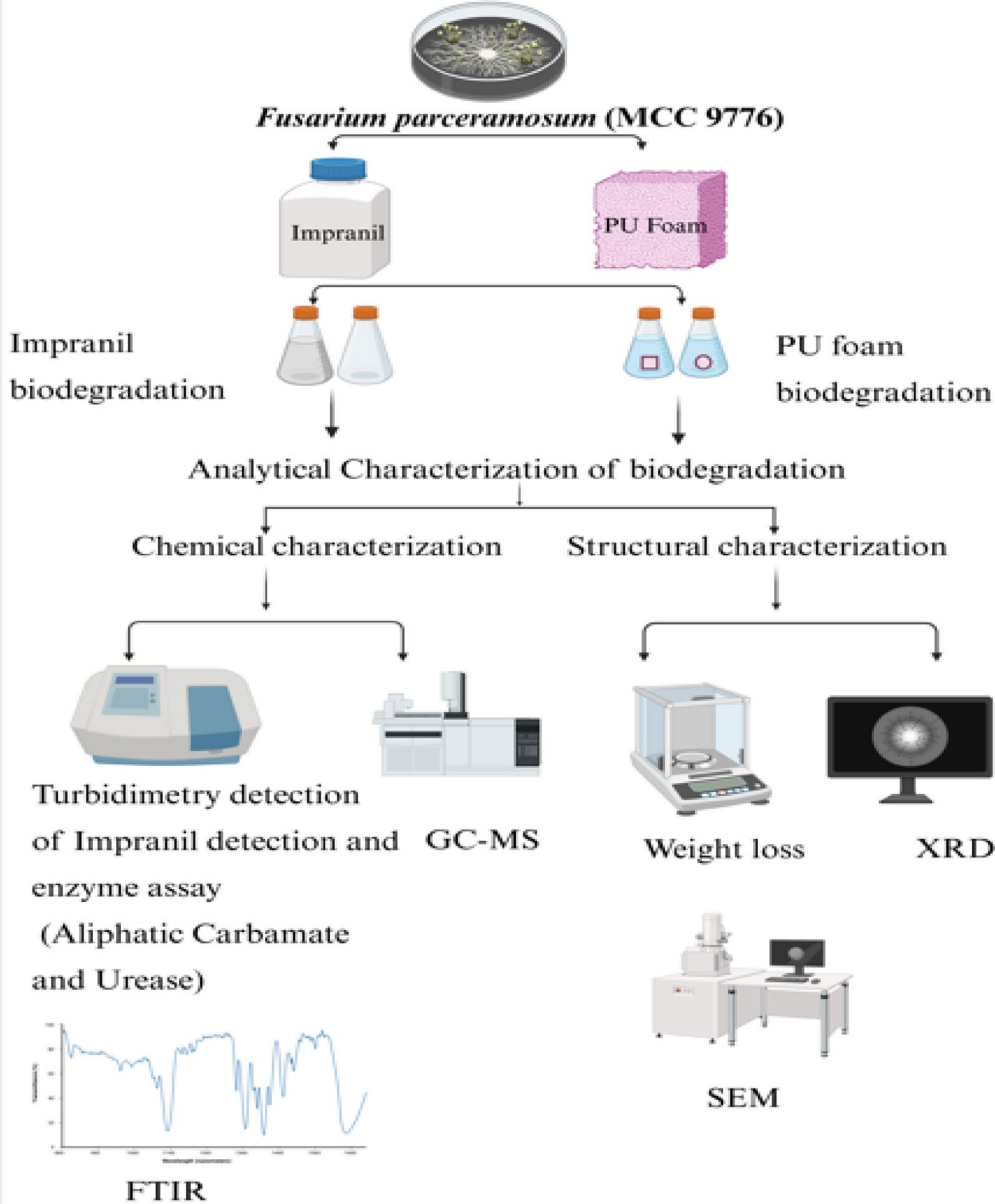

## Introduction

Polyurethane (PU) is an industrially versatile polymer formed by an exothermic reaction between two reactive chemical compounds (diisocyanates and polyols) (Zhang et al. 2025). The diverse industrial adaptability of this polymer has led to the accumulation of considerable amount of waste generated by it. Current approaches for PU waste management involves either landfilling or incineration, both of which are environmentally hazardous (Sahu et al. 2025).

Recycling methods have been explored, yet chemical recycling remains challenging due to emission of hazardous gases (Zhang et al. 2022). To overcome these limitations, recent studies have focused more on enzymatic degradation of PU, where microbial enzymes facilitate the breakdown of the polymer into non-toxic degradation products (i.e. CO_2_ and H_2_O), thereby reducing the ecological impact associated with conventional waste management practices (Nanda and Berruti 2021; Dey et al. 2024). PU is a chemically recalcitrant polymer, and its biodegradation is largely governed by its structural and chemical framework. Based on the chemical structure PU are broadly categorized as polyester (PS) and polyether (PE) PUs. PS-PUs are more prone to biodegradation as their polymeric backbone contains ester bonds that are more vulnerable to enzymatic hydrolysis, initiating polymeric fragmentation, biological assimilation and conversion of its secondary products. In contrast, PE-PUs have ether bonds in their polymeric backbone, making them chemically more resistant to enzymatic hydrolysis. In addition, to chemical framework, the relative proportion of hard and soft segments also influence the rate of biodegradation. Soft segments, derived from polyols, are more prone to enzymatic attack, whereas hard segments from isocyanates and chain extender groups increase structural stability and provides resistance to degradation. Also specific additives such as starch and bioactive extracts enhance microbial accessibility and enzyme susceptibility, thereby accelerating polymer degradation (Rajan et al. 2024; Sahu et al. 2025). While polymer chemistry and segmental composition govern susceptibility to degradation, the extent of biodegradation ultimately depend on the metabolic potential of microorganisms. In this process polymeric breakdown by the microbial groups is facilitated by effective enzymatic hydrolysis (M Bhuyar, 2018; Bhuyar et al. 2021). Extracellular enzymes facilitates more efficient biodegradation of PU, and are mainly secreted by fungal species (Jin et al. 2022). Along with the secretion of extracellular enzymes hyphal penetration and biofilm formation by fungal species on the polymeric surface enhances microbial attachment. In addition to enzymatic breakdown, fungal species also facilitates non-enzymatic breakdown of PU by secreting organic acids and secondary metabolites, surface erosion caused mainly due to reactive oxygen species (ROS) also helps in polymeric fragmentation, by enhancing polymeric accessibility for biocatalytic breakdown.

Fungal genera such as *Candida*, *Trichoderma*, *Penicillium*, and *Fusarium* synthesise quorum-sensing molecule (e.g., farnesol, tyrosol, lactones) that regulate hydrolase gene expression and mediate biofilm development. The integration of these enzymatic and non-enzymatic strategies across multiple fungal taxa underscores their functional relevance in accelerating PU biodegradation and advancing sustainable recycling processes (Okal et al. 2025).

In most studies, the initial evaluation of microbes, for their PU degradation potential, is mostly done using Impranil, (an aliphatic PS-PU dispersion) (Russell et al. 2011; Khruengsai et al. 2022). It is the most sensitive and convenient approach, where screening is done on the basis of optical changes associated with hydrolysis of this colloidal compound. However, the chemistry of Impranil substantially differ from other commercial available PU substrate. Therefore, for reliable assessment of microbial degradation there must be an intergradation of complementary analytical techniques (Magnin et al. 2019).

While PU biodegradation has been extensively explored using different microorganism, however studies demonstrating dual substrate specificity: the ability to degrade two chemically different PU substrates remains limited. While such capabilities have been extensively explored in bacterial systems, fungal studies are largely restricted to genera such as *Cladosporium* and *Aspergillus* (Álvarez-Barragán et al. 2016; Khan et al. 2020; Liu et al. 2023; Rajan et al. 2024; Maestri et al. 2026), where substrate specific degradation has been observed across different PU forms, including Impranil, solid foam, and PU films. Within *Cladosporium genus* substrate-specific degradation has been observed within species level, where *Cladosporium pseudocladosporioides* degrades Impranil, while *Cladosporium tenuissimum* degrades solid PU foam, indicating limited substrate specificity (Álvarez-Barragán et al. 2016). Although, *Cladosporium* sp.* P7* has been reported to degrade both Impranil and solid PU foam (Liu et al. 2023); however , such reports are limited, and studies demonstrating dual substrate specificity remain largely confined to a few well-characterized species. In addition, PU degradation under seawater conditions has been demonstrated using the marine-derived fungus *Cladosporium oxysporum*, which shows high efficiency in Impranil degradation and the ability to hydrolyse both ester and urethane bonds. The study further highlighted that enhanced microbial adhesion, achieved using chitosan nanoparticles, significantly improved degradation efficiency, emphasizing the importance of enzyme accessibility and microbe–substrate interactions in polyurethane biodegradation (Zeng et al. 2026). Additionally, species of *Fusarium* genus have been previously reported for PU biodegradation (Ren 2021; Okal et al. 2025), these studies are predominantly limited to PU films, which differ significantly in chemical composition and structural complexity from substrates such as Impranil and PU foam. Impranil is a colloidal PU dispersion with higher bioavailability, whereas PU foam is a highly crosslinked and more recalcitrant polymeric structure compared to PU films, and is more representative of real-world PU waste due to its widespread industrial and commercial applications. The present work highlights the ability of *Fusarium parceramosum* to degrade two chemically different PU substrates under unoptimized conditions, reflecting its dual substrate specificity within single fungal systems. This study provides the first evidence of PU biodegradation by *Fusarium parceramosum*, thereby contributing to the diversity of PU degrading fungi reported so far. *Fusarium parceramosum* was able to degrade both Impranil (a PU dispersion), and solid PU foam, highlighting its ability to act on PU in different physical forms and highlighting its dual-substrate degradation potential. This indicates that *Fusarium parceramosum* can act on different PU substrates, supporting its potential for wider use in biodegradation. The experiments were performed under unoptimized conditions to examine the inherent PU degradation ability of *Fusarium parceramosum* without external enhancement offering clearer understanding of its intrinsic ability to act on PU substrates. This approach enables the assessment of baseline activity and provides insight into the organism’s natural degradation potential. While optimization of parameters such as pH , temperature, and nutrient conditions may further enhance degradation efficiency, the present results reflect the intrinsic ability of *Fusarium parceramosum* to act on PU substrates. In addition, this work presents a detailed understanding of PU biodegradation by combining gravimetric measurements, quantitative evaluation of Impranil biodegradation, kinetic modelling, and advanced analytical techniques such as SEM, FTIR, XRD, and GC-MS, these analysis collectively highlight alterations in the polymeric structure, chemistry, and morphology. Based on the intermediates detected by GC-MS, a putative degradation pathway was proposed, suggesting their possible involvement in microbial metabolic process. Overall this combined approach helps in better understanding of fungal biodegradation of PU and highlights its role in sustainable waste handling, efficient resource use, and environmentally responsible ways to reduce persistent polymer pollution. It aligns with sustainable development goals (SDG) 12, (Responsible Consumption and Production) by enabling eco-friendly degradation of persistent PU waste and promoting circular resource use. It supports SDG 13 (Climate action) by reducing reliance on incineration and lowering pollution-association greenhouse gas emissions. Furthermore, it contributes to SDG 14 (Life Below Water) by preventing PU accumulation in aquatic systems and SDG 15 (Life on land) by mitigating soil contamination and protecting terrestrial ecosystem health through sustainable biodegradation strategies.

## Materials and methods

### Chemicals and reagents

Impranil DLH was obtained from Covestro AG, Germany, Yeast extract and Czapek Dox agar were obtained from HiMedia, India. Phenol, Sodium hydroxide, disodium hydrogen phosphate, and urea were procured from Loba chemicals, India. Sodium nitroprusside was obtained from Merck, India. Ethyl carbamate was procured from Spectrochem, India. Unless otherwise specified, all other chemicals and solvents were of analytical grade and procured from standard commercial suppliers.

### Experimental strain details

The fungal strain used in the study was previously isolated and identified as *Fusarium parceramosum* (Hegde et al. 2025). Identification was carried out by internal transcribed spacer (ITS) rRNA sequencing at National Centre for Microbial Resource (NCMR), Pune, India. The ITS sequence has been deposited in GenBank under accession number OM103045, and the culture has been deposited at NCMR under accession number MCC 9776.

### Initial screening of microorganism

For initial screening of PU-degrading ability of *Fusarium parceramosum*, Impranil containing agar plate was prepared using mineral salt media (MSM). MSM was prepared with the following composition: 0.5 g/L of dipotassium phosphate (K_2_HPO_4_), 0.2 g/L of ammonium sulfate ((NH_4_)_2_SO_4_), 0.1 g/L of sodium chloride (NaCl), 0.04 g/L of monopotassium phosphate (KH_2_PO_4_), 0.02 g/L of magnesium sulfate heptahydrate (MgSO_4_.7H_2_O), 0.002 g/L of calcium chloride dihydrate (CaCl_2_.2H_2_O), and 0.001 g/L of ferrous sulfate monohydrate (FeSO_4_.H_2_O). The medium pH was adjusted to 7.0 using NaOH/ HCl (Rajan et al. 2024). The MSM agar was sterilized at 121 °C for 15 min, and, after cooling, Impranil DLH was aseptically incorporated to a final concentration of 0.1% (v/v). Post-autoclave supplementation ensured that the polymer was not heat-degraded during sterilization. A loopful of actively growing *Fusarium parceramosum* culture was spot-inoculated onto MSM-Impranil agar plate, and incubated at 28 °C for 5 d, to assess its ability to grow in the presence of Impranil as the sole carbon source.

### Inoculum preparation

To obtain actively growing cultures, *Fusarium parceramosum* was freshly cultured on czapek dox agar plates at 28 °C for 5 d. Following incubation, a loopful of actively growing culture was aseptically transferred into Erlenmeyer flasks containing PU foam in MSM media supplemented with yeast extract. For Impranil degradation studies, two 1 cm^2^ agar plugs of actively growing *Fusarium parceramosum* on czapek dox agar plates, were excised aseptically and inoculated into 100 mL MSM containing various concentrations (0.1, 0.5, and 1%) of Impranil.

### Impranil biodegradation study

Impranil DLH (Covestro AG, Germany) was used as a model PU substrate. It is an anionic aliphatic PS PU dispersion supplied as white, low-viscosity aqueous systems with a solid content of approximately 40%. The polymer contains characteristic carbamate linkages (–NH–CO–O–) formed through the reaction of isocyanates and polyols, resulting in a segmented structure comprising hard and soft domains. The material is free from organic solvents and emulsifiers. The dispersion has a density of approximately 1.1 g/cm^3^. The dispersion remains stable in aqueous media over a broad range of pH and temperature conditions, making it suitable for biodegradation studies. Although the exact formulation is proprietary, it may contain additives such as stabilizers and other formulation components that contribute to its physicochemical stability. During degradation studies, a decrease in absorbance was monitored, which reflects a reduction in turbidity resulting from enzymatic breakdown of the polymer.

Impranil biodegradation was carried out in 100 mL Erlenmeyer flasks containing 50 mL of MSM medium. The medium was sterilized at 121 °C for 15 min, and after cooling, Impranil DLH was aseptically added to obtain final concentrations of 0.1, 0.5, 1% (v/v). Two agar plugs (1 cm^2^) of actively growing culture were aseptically excised and inoculated into 100 mL Erlenmeyer flasks containing MSM with desired Impranil concentrations. Control flasks consisting MSM and Impranil were also maintained under identical conditions.

All flasks were incubated at 28 °C and 100 rpm for 8 d. At every alternate day interval, 1 mL of culture sample was aseptically withdrawn and centrifuged. The resulting cell-free supernatant was analysed spectrophotometrically at 400 nm using UV–Visible spectrophotometer (Thermoscientific, Genesys 180) to determine residual Impranil concentration, with MSM serving as spectrophotometric blank (Peng et al. 2014). Residual Impranil concentration was quantified based on a standard curve prepared from known Impranil concentrations. Percentage degradation of Impranil was calculated using the formula:

1 $$ \% \;Degradation = \frac{{A_{0} - A_{t} }}{{A_{0} }} \times 100 $$

where A_0_ = Absorbance at 0th h, A_t_ = Absorbance at time t.

Following 8 d of incubation, the cell-free supernatant was collected and subjected to enzymatic assay to detect extracellular urease and aliphatic carbamate-hydrolyzing activities. All experiments were performed in triplicates, and the results are presented as mean ± standard deviation (SD).

### PU foam biodegradation study

PU foam (Toluene diisocyanate, TDI-based) was procured from local market in Manipal, Karnataka, India, and represents a flexible PU foam typically associated with polyester-based PU systems. The material consists of crosslinked polymer chains containing characteristics carbamate linkages (–NH–CO–O–) formed from the reaction of polyols and diisocyanates, resulting in a segmented structure with hard and soft domains. It exhibits a three-dimensional porous cellular architecture, providing a high surface area and flexibility, which influences its interaction with microbial systems during degradation studies. PU foams may also contain additives such as stabilizers, blowing agents, and other formulation components; certain additives can enhance microbial accessibility and enzyme susceptibility, thereby influencing polymer degradation. However, the exact composition of the material used in this study is not specified. The PU foam was subsequently cut into uniform cubes$(1.5 \times 1.5 \times 1.5\;cm$). Four PU foam cubes, were weighed prior to inoculation and transferred into 250 mL Erlenmeyer flask containing 100 mL of MSM supplemented with yeast extract (0.5 g/L). The flasks were sterilized at 121 °C for 15 min. The experimental sets were inoculated by aseptically adding a loopful of actively growing culture into each flask. Uninoculated flask containing only MSM and substrate (PU foam) served as the control. Four experimental sets were incubated at 28 °C with agitation at 150 rpm. Samples for analysis were collected from each set on the 7th, 30th, 50th, and 60th d of incubation. At each sampling interval, culture supernatants were collected for enzyme assay to detect extracellular urease and aliphatic carbamate-hydrolyzing activities. PU degradation was analytically characterised using Gravimetric analysis, Scanning electron microscopy (SEM), Fourier transform infrared spectroscopy (FTIR), X- ray diffraction (XRD), and Gas chromatography and mass spectroscopy (GC–MS). All experiments were performed in triplicates, and the results are presented as mean ± standard deviation (SD).

## Analytical methods

### Gravimetric weight loss

PU degradation was assessed by determining the weight loss of PU foam cubes over defined incubation intervals. Prior to inoculation, the combined weight of PU foam cubes were measured as initial weight (W_i_). At each sampling interval, the cubes were recovered, washed to remove adherent fungal biomass with 1% (w/v) sodium dodecyl sulfate (SDS) in bath sonication, followed by 3–4 cycles of washing with distilled water and a final immersion in ethanol. Washed cubes were dried at 50 °C for 24–48 h, and a final dry weight (W_f_) was determined. The percentage weight loss was calculated using Eq. 1. (Zeng et al. 2024):

2 $$ Weightloss\;\% = \frac{{W_{i} - W_{f} }}{{W_{i} }} \times 100 $$

### Kinetic modelling for PU foam and impranil biodegradation

Kinetic modelling of PU biodegradation was performed for Impranil (1% concentration) and PU foam using zero-order, first-order, Langmuir, and Freundlich models. Impranil at 1% concentration was selected for analysis as it represents the highest substrate concentration evaluated, enabling assessment of degradation behaviour under maximum substrate load. The kinetic parameters were estimated using regression analysis, and the goodness of fit of each model was determined based on the co-efficient of determination (R^2^). The degradation profiles were expressed as % degradation; determined as weight loss for PU foam and biodegradation % for Impranil, over incubation time.

The zero-order kinetic model assumes a constant degradation rate independent of substrate availability and is expressed as:

3 $${C}_{t}= {K}_{0} t$$

where, C_t_ represents the residual substrate at time t, expressed in percentage based on degradation and weight loss, C_0_ denotes the initial value, K_0_ is the zero-order rate constant, and t is the incubation time.

The first-order kinetic model assumes that the degradation rate is proportional to the concentration of the remaining substrate and is given by:

4 $${C}_{t}={C}_{0}{e}^{-kt}$$

where k is the first-order rate constant. The half life (t ½) was calculated using:

5 $${t}_{1/2}= \frac{ln2}{k}$$

The Langmuir model was applied to describe substrate-microbial interactions and to estimate degradation capacity and affinity constants:

6 $$C= \frac{{C}_{0}}{\left(1+kt\right)}$$

The Freundlich model was used to describe the empirical relationship between substrate degradation and microbial activity:

7 $$C=K {t}^{n}$$

### Enzyme assay

Urease and aliphatic carbamate-hydrolyzing activities were determined spectrophotometrically using the phenol-hypochlorite method with slight modifications of the procedure described by (Oceguera-Cervantes et al. 2007; Fuentes-Jaime et al. 2022). Cell-free supernatants, obtained by centrifuging culture broths at 10,000 rpm for 10 minutes at 4 °C, were utilized as crude enzyme sources. For each assay, 500 μL of supernatant was mixed with 250 μL of the respective substrates i.e. 5 M urea for urease and 5 M ethyl carbamate for aliphatic carbamate, and incubated at 50 °C for 3 min. Following incubation, 20 μL of the reaction mixture was transferred to a new tube, diluted with 2.23 mL distilled water, and sequentially mixed with 250 μL of phenol reagent and 500 μL of hypochlorite reagent. The mixture was incubated at 50 °C for 20 min to allow colour development, and absorbance was measured at 636 nm. Enzyme activities were quantified against ammonium chloride standard curves, and protein concentration in the supernatants were determined using Lowery’s assay at 660 nm (Lowry et al. 1951).

#### SEM

To examine surface changes in untreated and *Fusarium parceramosum* treated PU foam samples, SEM imagining was carried out. At every sampling interval PU foam (cubes) were washed thoroughly to remove fungal biomass and dried at 50 °C. Dried specimens were mounted on aluminum stubs using conductive carbon tape and sputter-coated with a thin layer of gold to enhance conductivity. Imagining was performed using Zeiss EVO18 scanning electron microscope (Model EVO 18, Carl Zeiss, Germany), and images of treated and untreated control foam were captured to compare structural integrity and document features associated with biodegradation.

#### FTIR

FTIR analysis was performed to evaluate alterations in functional groups present within the polymeric framework after 60 d incubation with *Fusarium parceramosum*. Spectra were recorded using Shimadzu spectrophotometer (Model 00254, Shimadzu Corporation, Japan) equipped with an ATR probe over the range of 4000 − 400 cm^− 1^. Treated and untreated samples were examined under identical conditions, and their characteristic peaks were compared. The difference between the two sets were compared to identify chemical alterations particularly linked to biodegradation.

#### XRD

Crystallinity of PU foam was analysed after 60 d of incubation using XRD analysis. Measurements were conducted using a Rigaku Mini Flex 600 X-ray diffractometer equipped with a Cu Kα radiation source (λ = 1.5406 Å). The instrument was operated at 40 kV and 15 mA in θ–2θ configuration. Diffraction patterns were collected over a 2θ range of 5–90°, with a step size of 0.01° and a scan speed of 2°/min. Both control and treated samples were analysed under identical conditions. Comparative evaluation of the diffractograms was carried out to assess alterations in crystalline domains and structural order induced by biodegradation. Percent crystallinity (XC) was calculated using Eq. 2. (Chen et al. 2024).

8 $$ XC\;\% = \frac{{A_{c} }}{{A_{c} + A_{a} }} \times 100 $$

where XC is % crystallinity, A_c_ is area under crystalline region, and A_a_ is area under amorphous region.

#### GC–MS

The degradation intermediates of PU foam and Impranil following fungal incubation were characterised by GC–MS analysis. For both PU foam and Impranil, culture supernatants from treated and control sets were analysed. Methanol was employed as the extraction solvent, and the processed samples were subjected to GC–MS analysis. The analysis was conducted on an Agilent 7890 GC system coupled with a 5977A mass selective detector, equipped with an Agilent DB-6 ms capillary column. The instrument was operated in electron ionization (EI) mode. Sample introduction was performed using an automated PAL sampler in split mode, with the inlet temperature maintained at 250 °C. Helium was used as the carrier gas, and the MSD transfer line was maintained at 280 °C. Data acquisition was performed in scan mode over an m/z range of 35–700. Mass spectral identification of the detected compounds was carried out by comparison with reference spectra from the NIST Mass Spectral Library.

### Statistical analysis

All experiments were conducted in triplicate and are expressed as mean ± standard deviation (SD). Statistical significance of the degradation efficiency was determined using one-way analysis of variance (ANOVA). A significance level of p-value < 0.05 was adopted for all analyses. Statistical analyses were performed using Microsoft Excel.

## Results

### Initial screening of microorganism

**Fig. 1:**
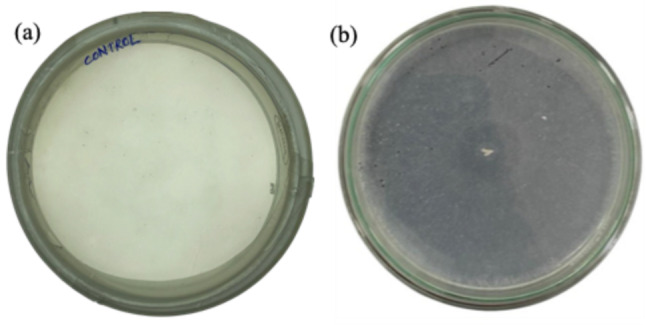
Initial screening of *Fusarium parceramosum* on MSM–Impranil plates, **a** Control plate, **b** experimental plate

After 5 d of incubation at 28 °C, *Fusarium parceramosum* produced a pronounced halo zone (Fig. 1), signifying hydrolysis of Impranil and confirming the ability of this fungus to utilize Impranil as a sole carbon source. In contrast, halo zone formation was not observed in the uninoculated control plates, suggesting that the formation of halo zone was specifically attributed to fungal metabolism. Visualizing the formation of halo zones on Impranil agar plates is a widely used method for initial screening of microorganism for their PU degrading capabilities (Rüthi et al. 2023).

### Impranil biodegradation

**Fig. 2:**
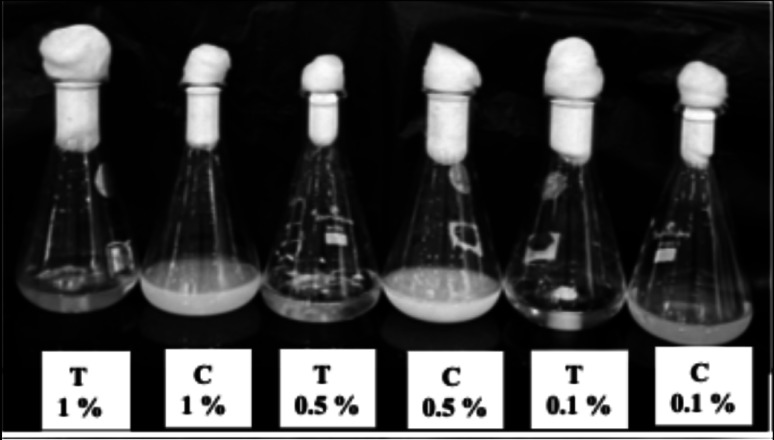
Impranil biodegradation across varying substrate concentrations in culture flasks. (C: Control, T: Test). All experiments were performed in triplicate and expressed as mean ± SD; statistical significance was determined using one-way ANOVA (*p* < 0.05)

**Fig. 3:**
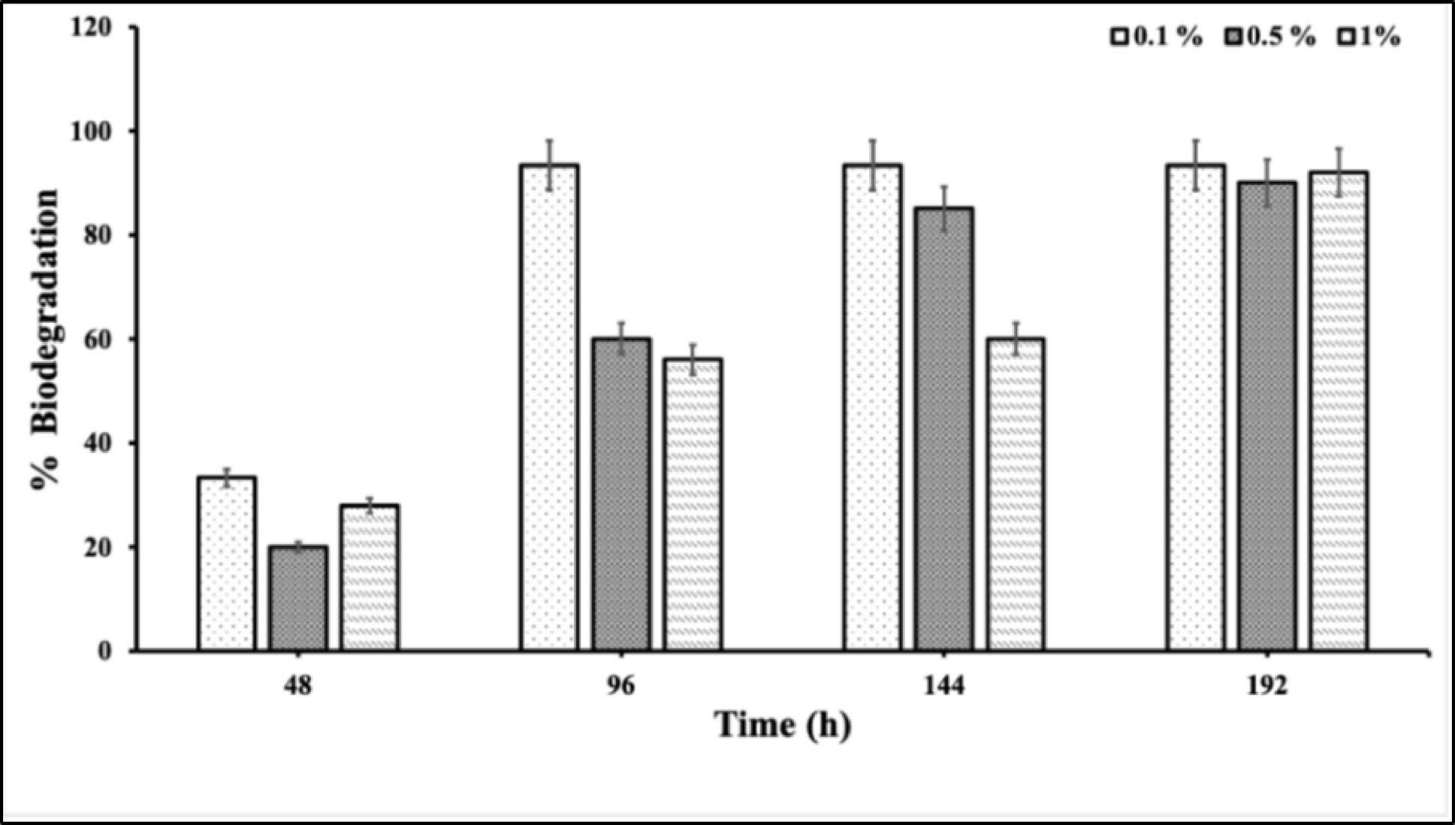
Impranil biodegradation using *Fusarium parceramosum.* All experiments were performed in triplicate and expressed as mean ± SD; statistical significance was determined using one-way ANOVA (*p* < 0.05)

The biodegradation of Impranil DLH by *Fusarium parceramosum* was quantitatively assessed in liquid culture at three initial concentrations (0.1, 0.5, and 1% v/v) over 192 h (8 d). Percentage degradation represents the reduction in absorbance at 400 nm relative to initial (0th h) absorbance. At 48 h, degradation was already apparent, with 33.3, 20, and 28% of 0.1, 0.5, and 1% Impranil concentrations respectively. Such initial onset of degradation aligns with the accelerated biocatalytic response, generally seen in fungal PU biodegradation studies (Khruengsai et al. 2022). By 96 h, the lower concentration 0.1% showed rapid substrate utilization, achieving 93.3% degradation, while 0.5% and 1% treatments exhibited 60% and 56% degradation, respectively. At 144 h, near-complete degradation of 93.3% was observed at 0.1% Impranil concentration; whereas, 85% and 60% degradation at 0.5 and 1% Impranil concentrations was recorded respectively. By 192 h, Impranil degradation was efficiently high across treatments at 0.1, 0.5, and 1% were 93.3, 90, and 92% respectively, demonstrating concentration-dependent degradation (Figs. 2 and 3). Overall the findings, confirms the biodegradation potential of *Fusarium parceramosum* in shake-flask culture conditions, with early degradation at minimal substrate concentrations.

### Gravimetric weight loss analysis

**Fig. 4:**
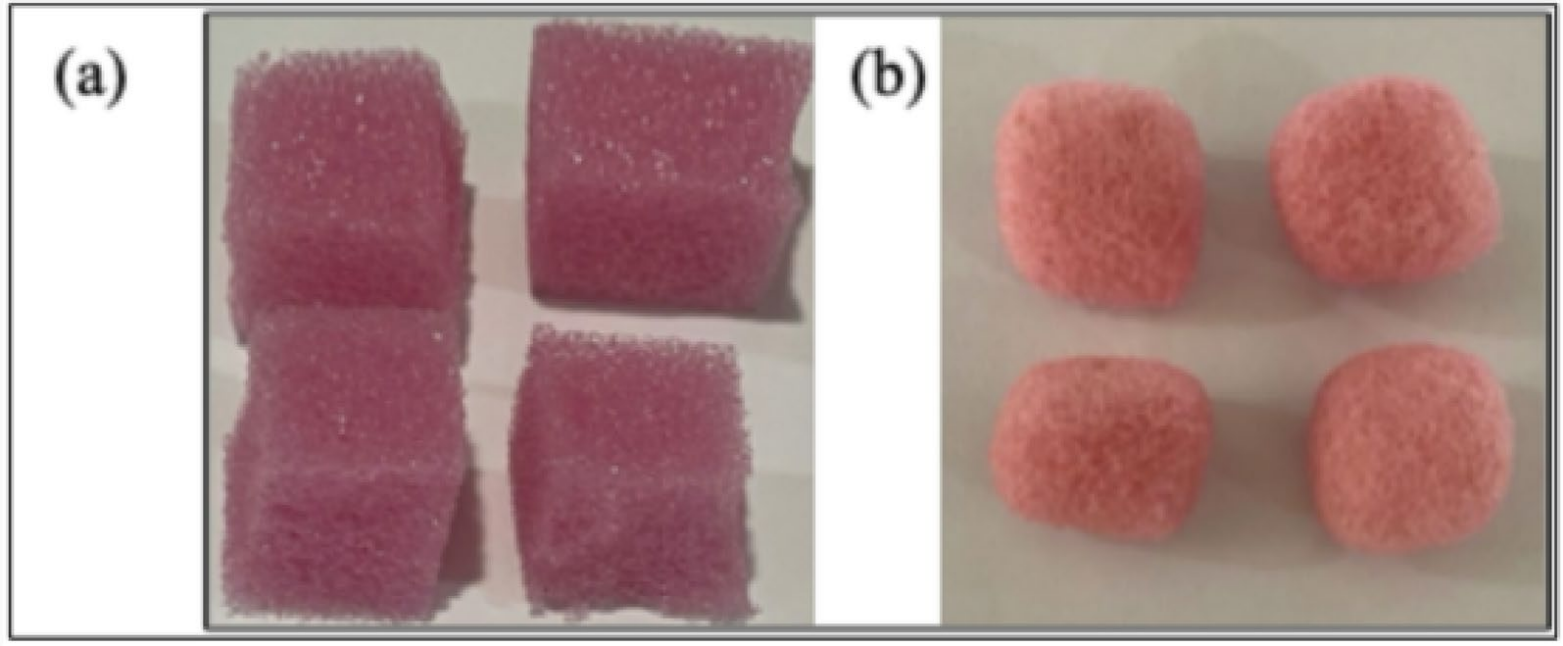
**a** PU foam without fungal treatment as control, **b** PU foam after 60 d of incubation with *Fusarium parceramosum*

**Fig. 5:**
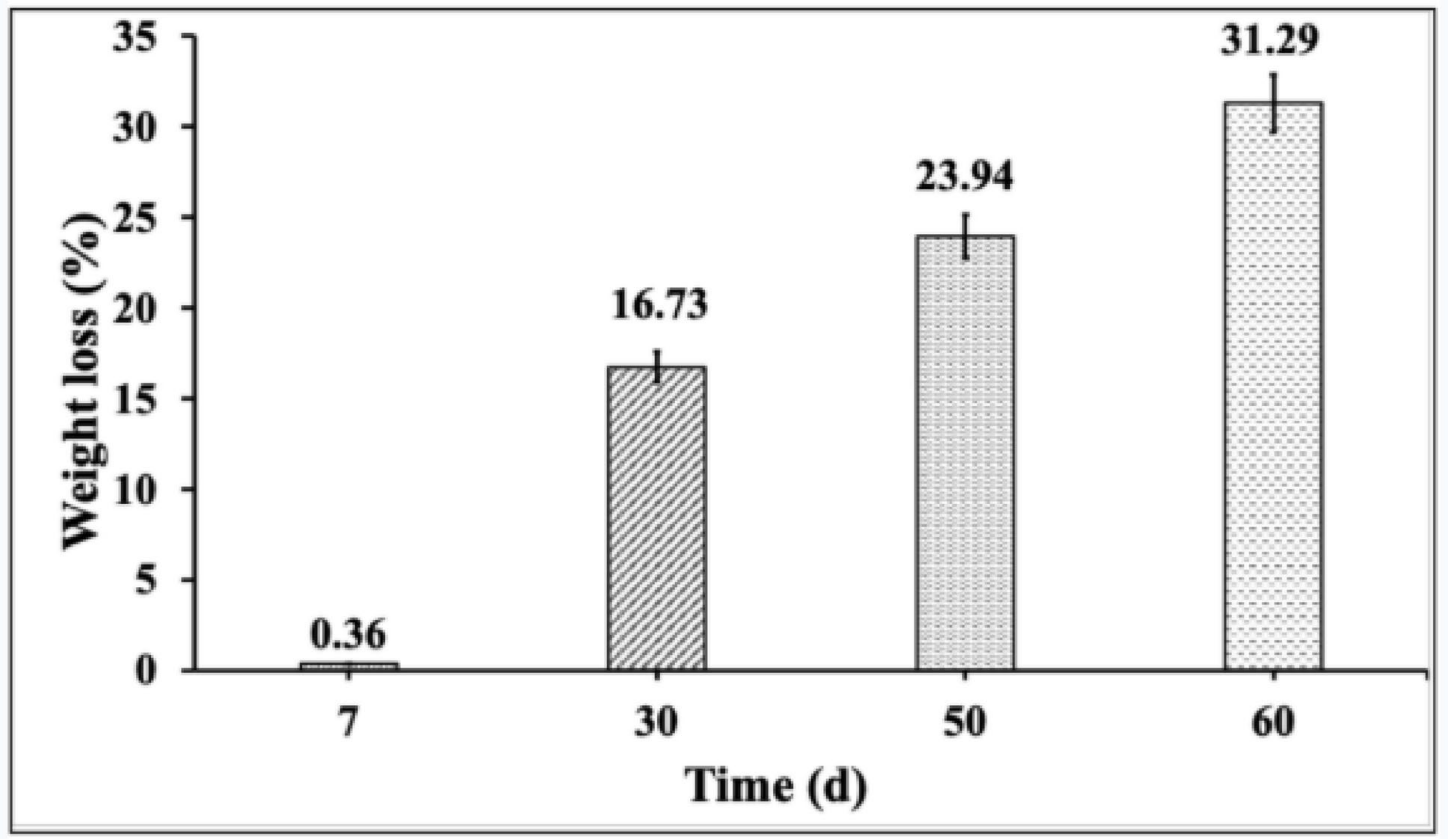
Gravimetric weight loss % of PU foam after treatment with *Fusarium parceramosum.* All experiments were performed in triplicate and expressed as mean ± SD; statistical significance was determined using one-way ANOVA (*p* < 0.05)

PU foam biodegradation by *Fusarium parceramosum* was evaluated through gravimetric measurements, by recording the cumulative weight loss % of PU cubes over 60 d incubation period. An initial negligible reduction of 0.36% was observed after 7 d, suggesting limited early colonization and enzyme activity. A gradual increase in degradation was recorded, with PU cubes showing 16.73% of weight loss by 30 d followed by 23.94% by 50 d. Maximum weight loss of 31. 29% occurred at 60 d. This gradual decrease in weight loss % highlights the sustained colonization and biodegradation efficiency of *Fusarium parceramosum* (Figs. 4 and 5), similar to observations reported in extended microbial degradation studies (Zhang et al. 2025).

### Kinetic modelling for PU foam and impranil biodegradation

The degradation data for PU foam and Impranil (1%) were fitted to zero -order, first-order, Langmuir, and Freundlich kinetic models. The corresponding kinetic parameters are summarized in Table. 1.

**Table 1:** Kinetic parameters and R^2^ values for PU foam and Impranil biodegradation using different models

Model	Equation	Parameters		R^2^	
		PU foam	Impranil	PU foam	Impranil
Zero-order	C = C_0_−Kt	K = 0.0015, C_0_ = 0.2684	K = 0.0001, C_0_ = 0.024	0.9984	0.9839
First -order	C = C_0_ e^−kt^	K = 0.007	K = -0.0105	0.9988	0.9827
Langmuir	C = C_0_/ (1 + Kt)	K = 0.0084	K = 0.0377	0.3737	0.8964
Freundlich (Power law)	C = Kt^n^	n = − 0.0982, K = 0.287	n = -0.0036, K = 1.027	0.6774	0.8755

**Fig. 6:**
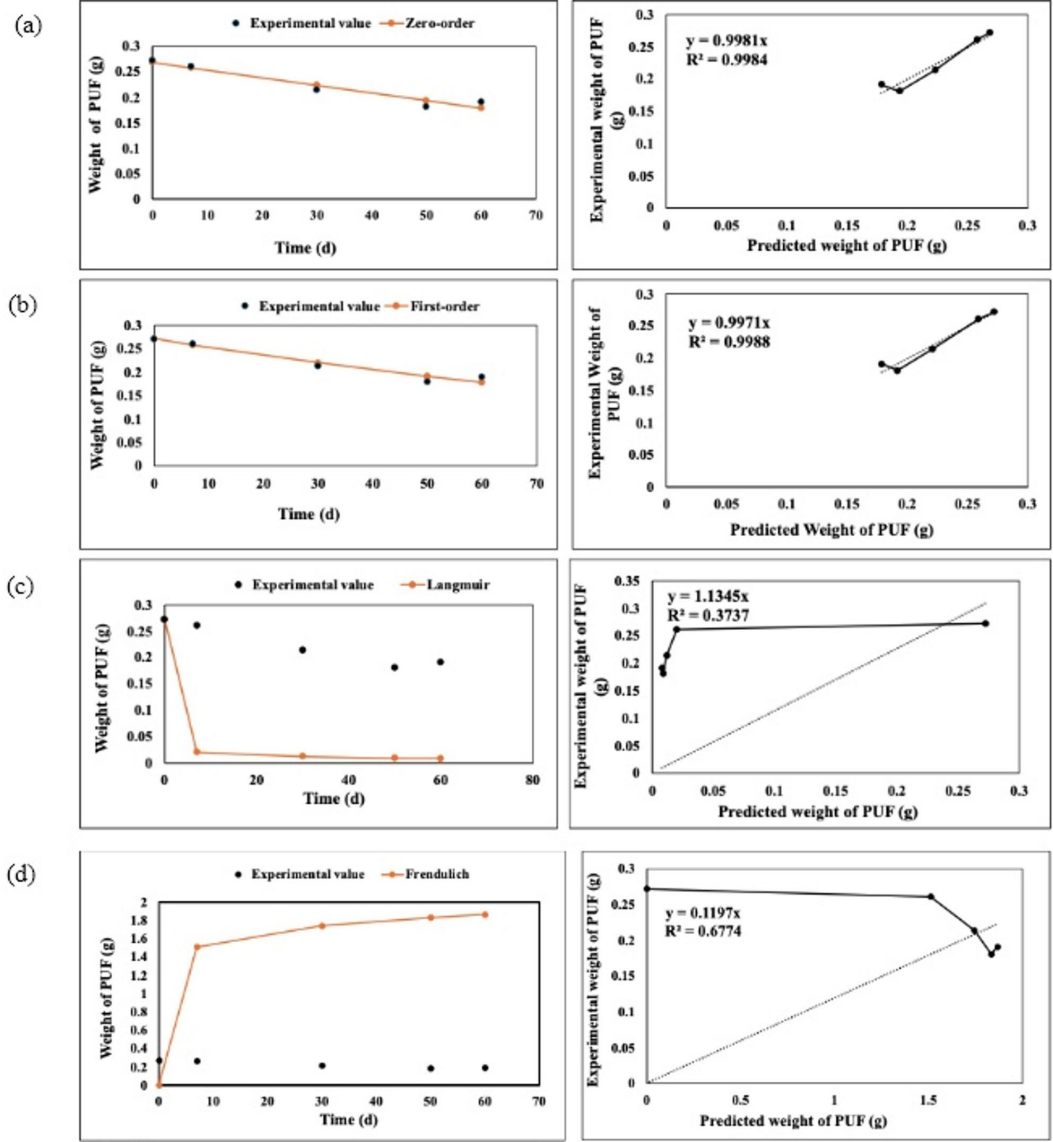
Modeling of polyurethane foam (PUF) degradation kinetics using zero-order **a**, First-order **b**, Langmuir **c**, and Freundlich models **d**, showing experimental data, predictions, and regression fits

For PU Foam, the first-order model showed the highest correlation (R^2^ = 0.9988), followed closely by the zero-order model (R^2^ = 0.9984). In comparison, the Freundlich (R^2^ = 0.6774) and Langmuir (R^2^ = 0.3737) models exhibited lowers fits (Fig. 6).

**Fig. 7:**
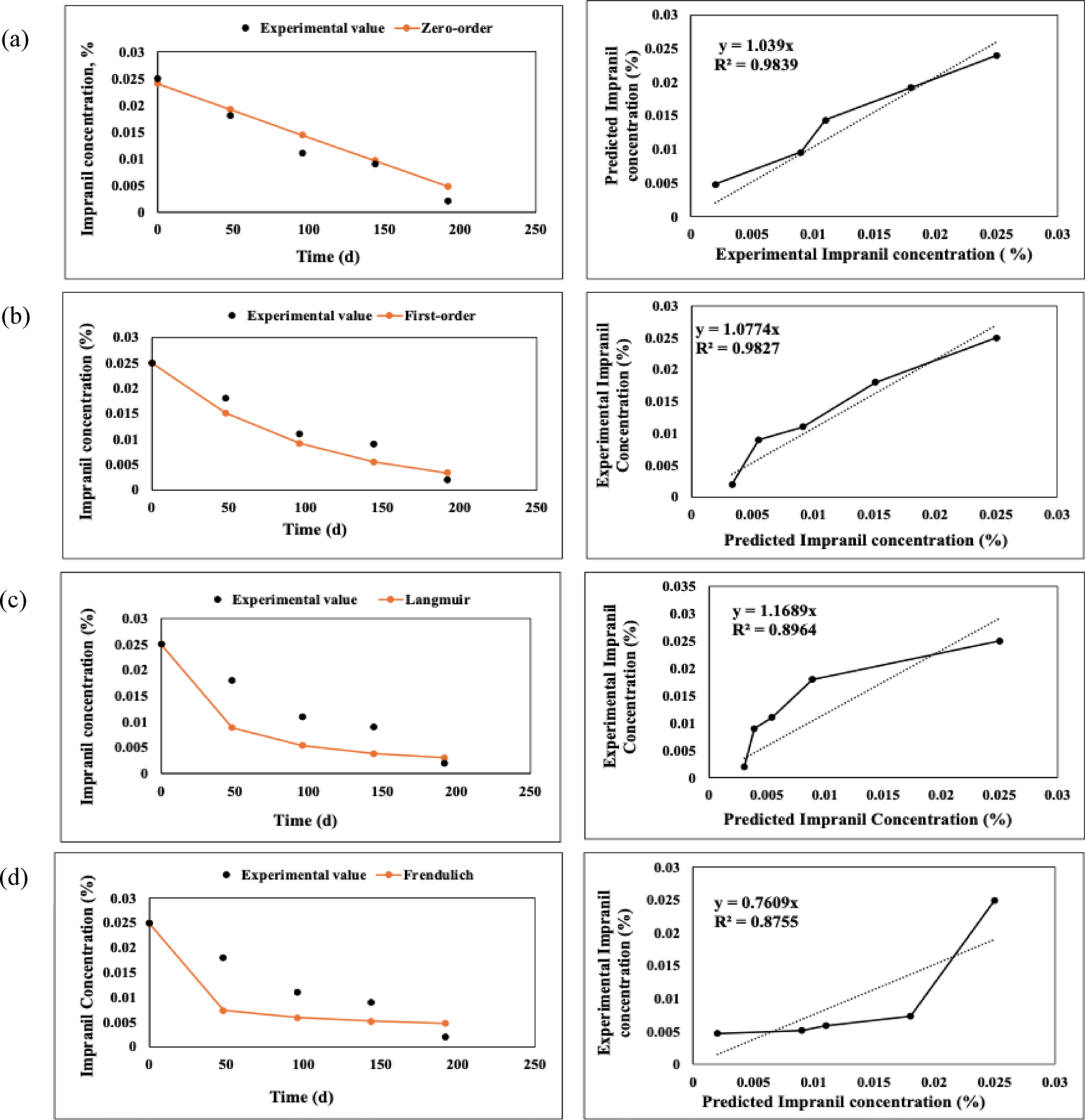
Modeling of Impranil degradation (1%) kinetics using zero-order **a**, First-order **b**, Langmuir **c**, and Freundlich models **d**, showing experimental data, predictions, and regression fits

For Impranil (1%), the zero-order model showed the highest correlation (R^2^ = 0.9839), closely followed by the first-order model (R^2^ = 0.9827). The Langmuir (R^2^ = 0.8964) and Freundlich (R^2^ = 0.8755) models showed comparatively moderate fits (Fig. 7)

### Enzyme assay

**Fig. 8:**
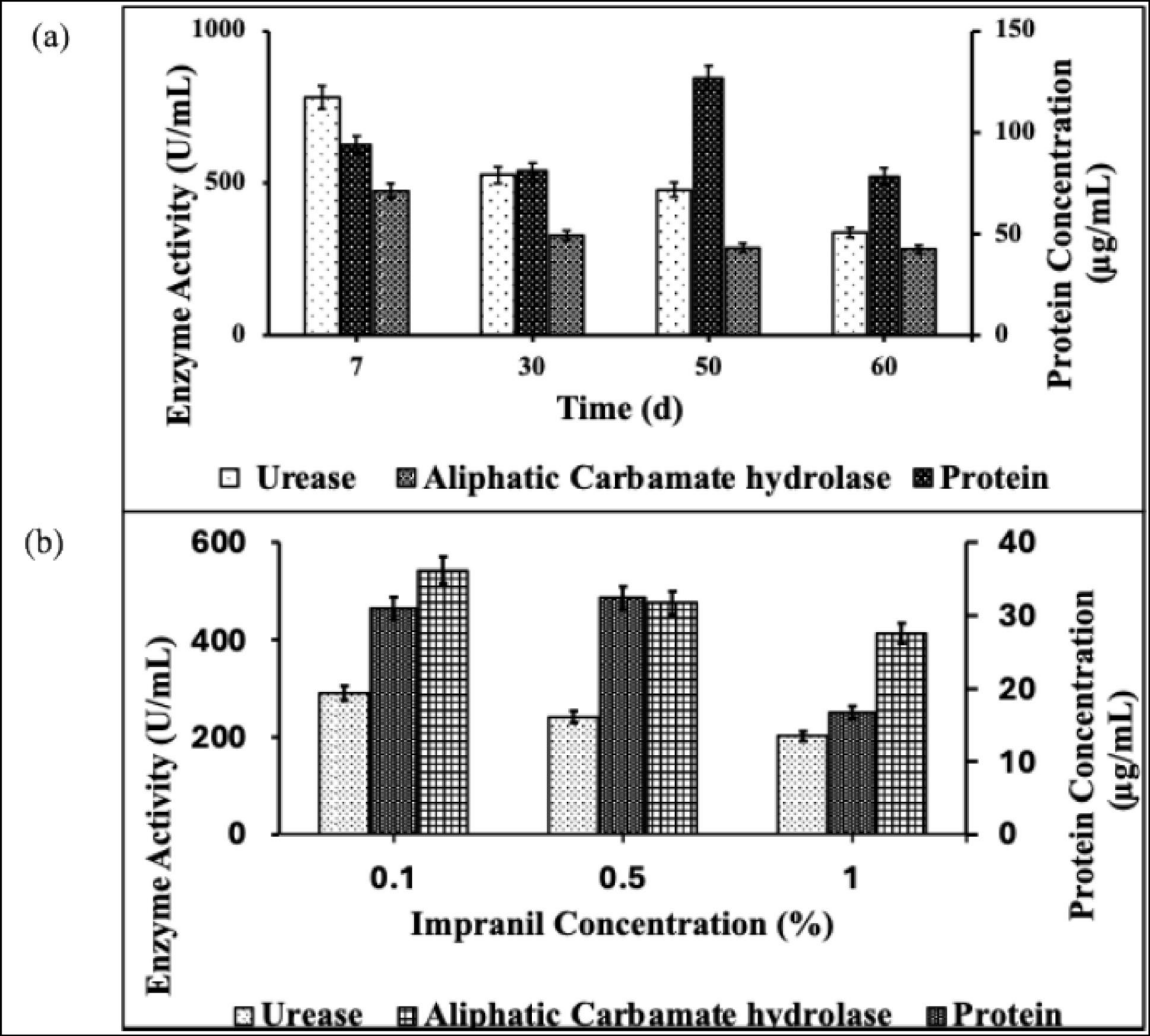
Urease and aliphatic carbamate-hydrolyzing activities of *Fusarium parceramosum* on **a** PU foam, and **b** Impranil. All experiments were performed in triplicate and expressed as mean ± SD; statistical significance was determined using one-way ANOVA (*p* < 0.05)

*Fusarium parceramosum* exhibited measurable urease and aliphatic carbamate-hydrolyzing activities throughout the 60-d incubation period with PU foam (Fig. 8a). Aliphatic carbamate-hydrolyzing activity showed a declining trend, decreasing from 475.41 U/mL on d 7 to 328.53 U/mL on d 30, 288.96 U/mL on d 50, and 284.17 U/mL on d 60. Similarly, urease activity demonstrated a consistent decline from 781.17 U/mL on d 7 to 526.97 U/mL on d 30, 478.41 U/mL on d 50, and 338.12 U/mL on d 60. Protein concentrations in crude enzyme extracts varied over time, with levels of 93.79 µg/mL at d 7, 81.01 µg/mL at d 30, peaking at 126.75 µg/mL at d 50, and declining to 78.51 µg/mL at d 60.

For Impranil biodegradation enzyme activities were measured for the treatments with different amounts of added Impranil (Fig. 8b). An inverse relationship with substrate concentration was observed in case of both the enzymes. Aliphatic carbamate-hydrolyzing activity increased from 412.47 U/mL at 1.0% Impranil to 476.61 U/mL at 0.5% and reached maximum activity of 543.16 U/mL at 0.1% concentration. Urease activity followed a similar pattern with 202.03 U/mL at 1.0%, 241.00 U/mL at 0.5%, and 290.76 U/mL at 0.1% of Impranil. Corresponding protein concentrations from Impranil treatments were 16.66 µg/mL at 1.0%, 32.40 µg/mL at 0.5%, and 31.01 µg/mL at 0.1% of substrate concentration. The temporal enzyme activity profiles and substrate concentration effects confirm the enzymatic capabilities of *Fusarium parceramosum* for PU biodegradation through aliphatic carbamate and urease production.

#### SEM

**Fig. 9:**
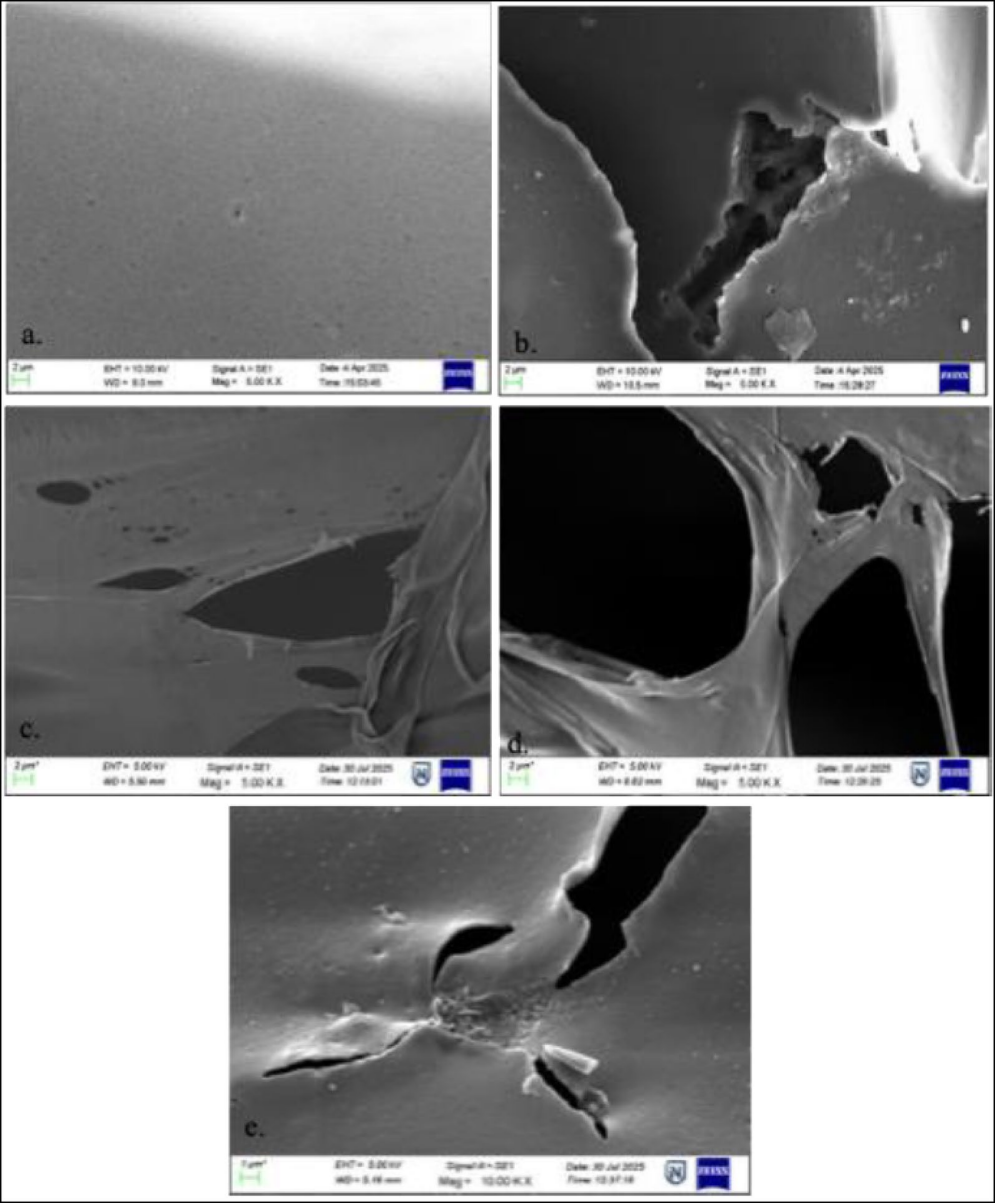
SEM images highlighting the changes on the surface of PU foam following incubation with *Fusarium parceramosum*

SEM analysis provided clear evidence of biodegradation in the *Fusarium parceramosum*-treated sample (Fig. 9). The untreated control (Fig. 9a) exhibited smooth intact polymer surface, indicating pristine nature of the polymer. After 7 d of incubation, surface cracks and minor irregularities became apparent (Fig. 9b). By the 30th d, surface tearing was observed (Fig. 9c), indicating initiation of polymeric breakdown. On the 50th d, large voids were prominently observed with visible surface thinning (Fig. 9d). After the 60th d, the surface deterioration became severe with deep cavities being clearly observed (Fig. 9e). These observations co-related with gravimetric weight loss data, together demonstrating the ability of *Fusarium parceramosum* to degrade PU foam over time.

#### FTIR

FTIR was used to detect variations in functional groups and modification of chemical bonds in PU foam, after 60 d incubation with *Fusarium parceramosum*, and is represented in Table 2.

**Table 2:** FTIR spectral changes observed in control and treated samples

Sl. no	Functional group	Control (cm^− 1^)	Treated (cm^− 1^)	Interpretation
1	N–H stretching	3285	3279	Slight shift, reduced intensity
2	Aliphatic C–H stretching	2971 − 2867	2973 − 2864	Minimal Shift
3	C= O stretching	1721	1718	Partial hydrolysis
4	C–O stretching	1093	1088	Limited degradation, reduced intensity
5	Nitrile / Isocyanate	–	2210	Weak new band, derivatives of biodegradation intermediates

**Fig. 10:**
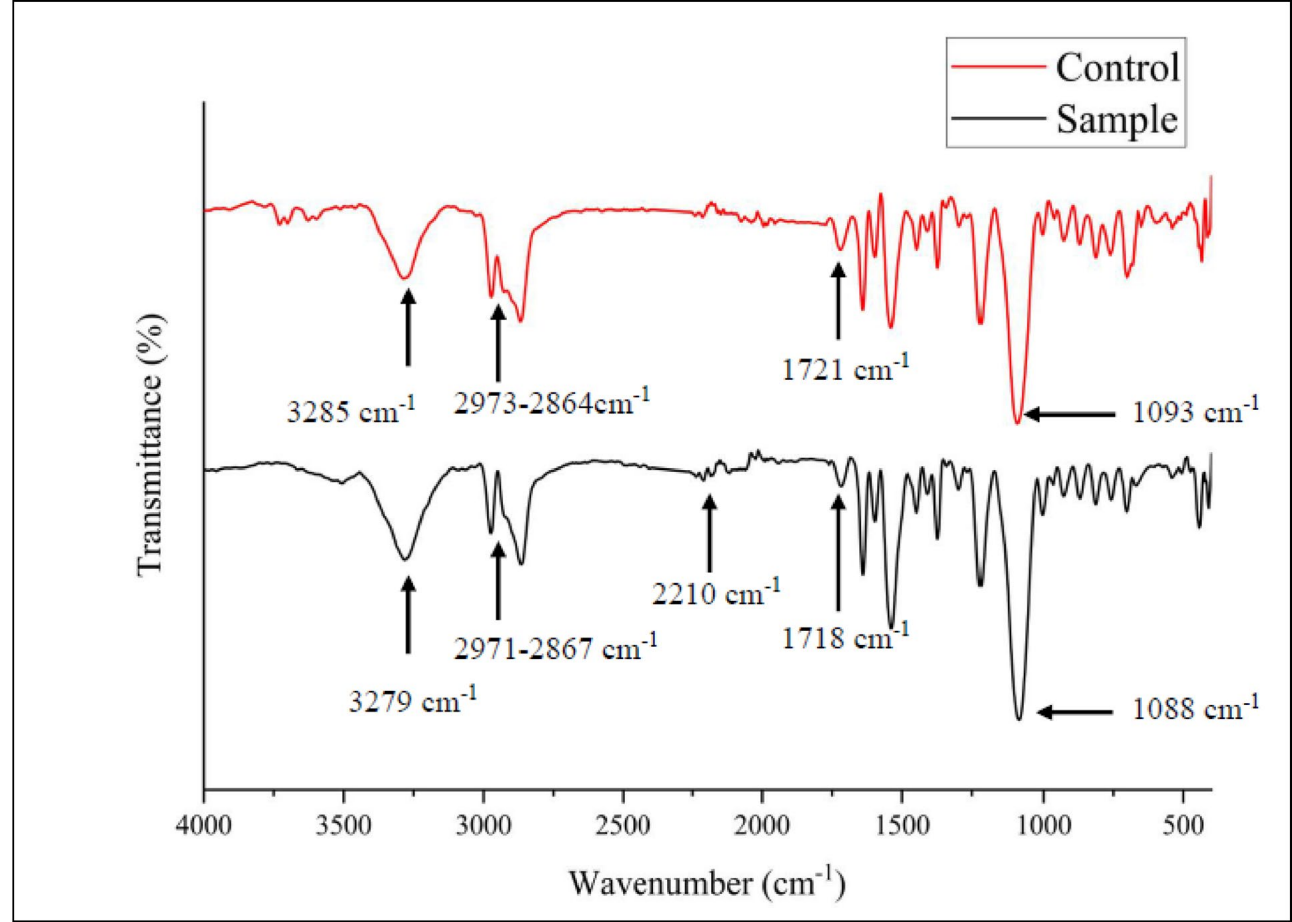
FTIR spectral comparison between the control and the *Fusarium parceramosum*-treated PU foam

The spectral shifts and emergence of new peak (Fig. 10) confirms the ability of *Fusarium parceramosum* to induce chemical modification within PU matrix, highlighting its biodegradation potential.

#### XRD

XRD analysis was used to detect difference in structural crystallinity between control and *Fusarium parceramosum*- treated PU foam. Significant crystalline peaks were present in control sample as compared to the treated one as shown in Table 3. While peak position persisted across both the samples, the decrease in intensity was prominent in the treated sample, indicating partial disruption of crystalline domains. Percentage crystallinity was calculated for both the control and the treated sample to quantitatively measure the loss in the crystalline domains upon fungal incubation, as shown in Table 3.

**Table 3:** XRD profiles of untreated and *Fusarium parceramosum* treated PU foam following 60 d incubation

Sl. no	Parameter	Control	Treated
1.	Crystalline peak (19.94°, 28.84°, and 29.88° 2θ)	Crystalline peak intensities 286, 343, 306 (a.u.)	Crystalline peak intensities 104, 171, 88 (a.u.)
2.	% Decrease in crystalline peak after biodegradation	–	61.2, 50.1, 71.2%
3.	Crystalline area (A_c)_	100,825	174,816
4.	Amorphous area (A_a_)	174,816	151,200
5.	% Crystallinity (XC)	36.6	13.4
6.	Interpretation	Semi-crystalline structure	Significant breakdown of crystalline domains caused by *Fusarium parceramosum*

**Fig. 11:**
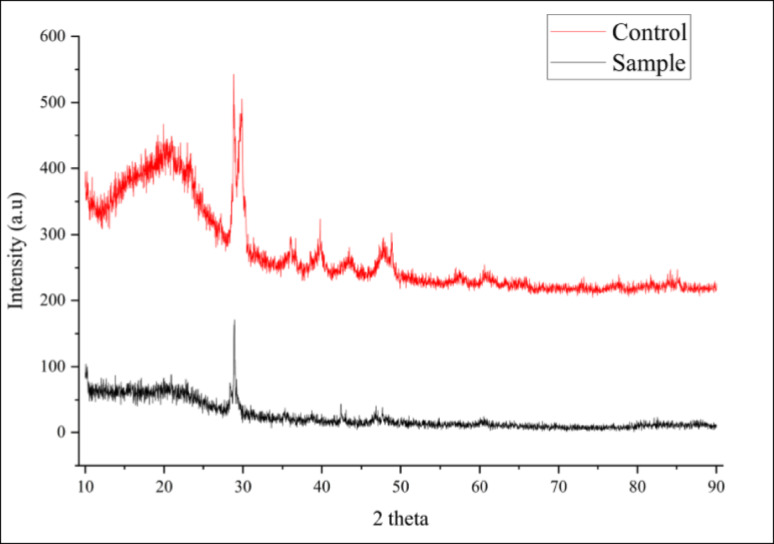
XRD patterns of untreated and *Fusarium parceramosum*-treated PU foam

These results indicate a marked reduction in crystalline domains of PU foam following 60 d of incubation with *Fusarium parceramosum* (Fig. 11)

#### GC–MS

**Fig. 12:**
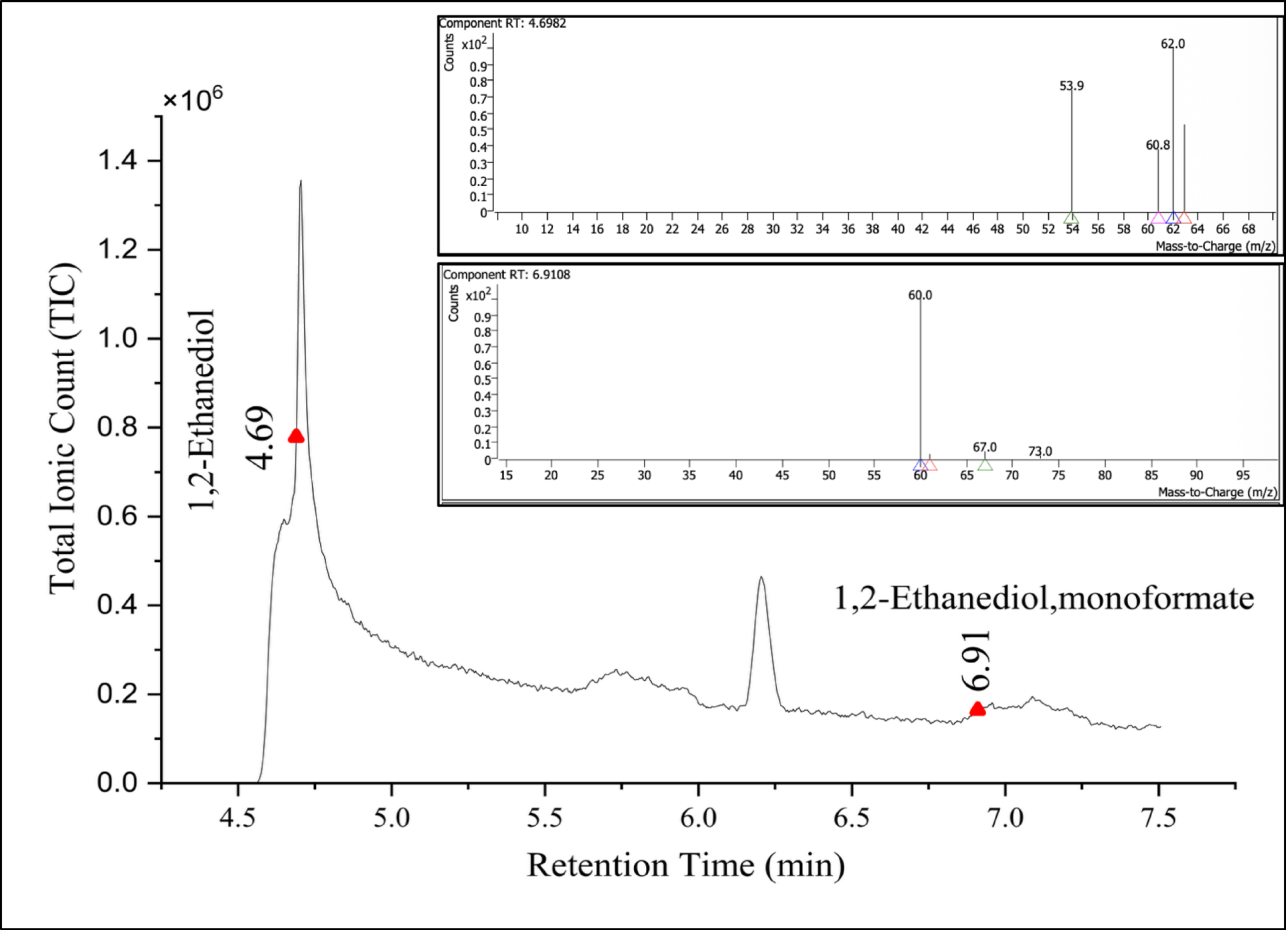
GC–MS chromatogram and mass spectra showing detection of 1,2-ethanediol and 1,2-ethanediol monoformate

**Fig. 13:**
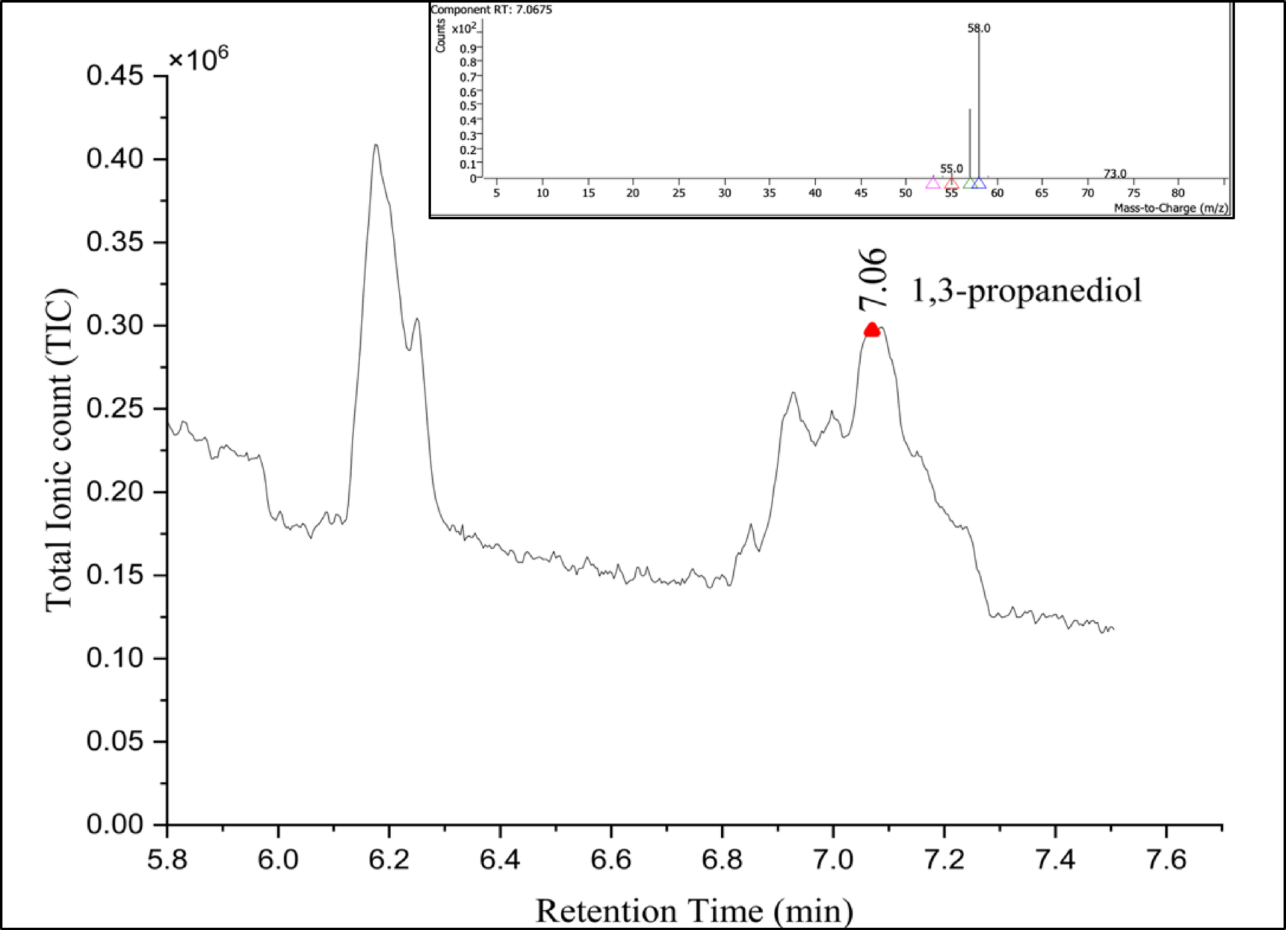
GC–MS chromatogram and mass spectra showing detection of 1,3-Propanediol

GC–MS profiling of the degraded PU foam and Impranil highlighted several degradation intermediates, indicating hydrolytic and oxidative degradation processes as shown in Table 4. Two specific diols 1,2-ethanediol and its oxidative derivative 1,2-ethanediol, monoformate were specifically detected in 60 d *Fusarium parceramosum* treated PU foam sample (Fig. 12). These intermediates were absent in the control and serve as robust chemical markers indicative of hydrolytic cleavage and subsequent oxidative transformation of polyether soft segments. Additionally, for 1% Impranil sample, 1,3-propanediol was specifically detected in the treated sample (Fig. 13) indicating breakdown of polyester segments soft segments. Together, the GC–MS data demonstrates that degradation of both solid PU foam and aqueous Impranil DLH dispersion results in the release of specific diol intermediates. The unique presence of 1,2-ethanediol, 1,2-ethanediol monoformate, and 1,3-propanediol in treated samples but not in controls provides direct evidence of polymer breakdown.

**Table 4:** GC–MS–identified intermediates generated during biodegradation of PU foam and Impranil DLH

Sl. No	Compound name	Retention time	Molecular formula	Peak area	Relevance in study	Substrate type
1	1,3-Propanediol	7.067	C_3_H_8_O_2_	551,460.08	Hydrolysis product of polyester segments, marker of Impranil degradation	Impranil DLH
2	1,2-Ethanediol	4.698	C_2_H_6_O_2_	56,490.30	Hydrolytic cleavage marker of polyether soft segments	PU Foam
3	1,2-Ethanediol, monoformate	6.910	C_3_H_6_O_3_	81,144.80	Oxidative transformation product of 1,2-ethanediol

## Discussion

This study highlights the dual substrate specific nature of *Fusarium parceramosum* towards two chemically different PU substrates (Impranil and PU foam). Degradation was systematically evaluated using gravimetric weight loss, enzymatic assay, and comprehensive physiochemical analyses. The ability of *Fusarium parceramosum* to utilize PU as a sole carbon source was initially evaluated on MSM agar plates supplemented with 0.1% Impranil (v/v). After 5 d of incubation, the formation of a clear halo zone indicated enzymatic hydrolysis of Impranil (Fig. 1). This method serves as a standard preliminary approach to evaluate the PU degrading capabilities of microorganisms (de Witt et al. 2024). In a recent study by (Giyahchi and Moghimi 2023), Impranil hydrolysis was visualized through halo zone formation, enabling the identification of two yeast strains NS-7 and NS -12 with PU degrading potential. The growth kinetics indicated that the maximum growth of NS- 7 occurred between the 8 d and the12 d of incubation, whereas growth of NS-12 peaked between the 4 d and the 6 d. In our study *Fusarium parceramosum* exhibited prominent growth by the 5 d (Fig. 1), followed by visible halo zone on MSM-Impranil plates, indicating rapid degradation activity of *Fusarium parceramosum* under the tested conditions.

While the formation of halo zone on Impranil agar plates provided preliminary evidence of PU degrading capabilities of microorganisms, quantitative evaluation is necessary to assess the actual degradation kinetics. Impranil degradation is monitored by reduction in optical density over the period of incubation. Microorganisms capable of utilizing Impranil as a sole carbon source eventually convert this opaque solution into a transparent one. This provides a real-time assessment of microbial degradation efficiency. However, reduction in turbidity (absorbance at 400 nm) may also be influenced by factors such as fungal biomass aggregation, adsorption of polymer particles onto fungal biomass, and sedimentation effects, and therefore not exclusively represents polymer degradation. In particular, adsorption of PU particles onto fungal hyphae can lead to their removal from the suspension phase, while biomass growth may promote aggregation and flocculation of particles, enhancing their settling. These processes can collectively contribute to an apparent decrease in turbidity independent of actual polymer breakdown. To minimize these potential interferences, a control without inoculum was included to account for non-biological changes in turbidity, and all measurements were performed under consistent experimental conditions. Additionally, OD 400 nm results were interpreted alongside complementary analytical evidence. In the present study, GC-MS analysis revealed the presence of 1,3-propanediol in treated samples, a known degradation intermediate associated with PU breakdown, thereby supporting that the observed turbidity reduction is associated with biodegradation.

In earlier work, (Pantelic et al. 2024) investigated the biodegradation of Impranil using bacterial isolates at substrate concentrations of 0.5 , 1, and 1.5% and demonstrated rapid degradation of 1% Impranil within 48 h. Expanding upon our preliminary observations, *Fusarium parceramosum* was screened for its biodegradation capabilities at different Impranil concentration ( 0.1, 0.5 and 1% v/v) by extending the observation period to monitor precise degradation kinetics (Figs. 2 and 3). Similarly, (Liu et al. 2023) achieved complete mineralization of Impranil under optimized conditions . While (Pantelic et al. 2024) reported early-stage degradation of Impranil by a bacterial isolate and (Liu et al. 2023) studied Impranil degradation under optimized conditions, the present study examined the degradation of Impranil by *Fusarium parceramosum* under fixed unoptimized conditions. The results highlight the potential of *Fusarium parceramosum* under fixed environmental conditions, which allows establishment of a baseline for further optimization.

Following Impranil dispersion assay, the current study also examined the degradation of solid PU foam by *Fusarium parceramosum*. The extent of PU foam biodegradation over 60 d incubation with *Fusarium parceramosum* was primarily assessed through gravimetric weight loss analysis, culminating in a maximum weight loss of 31.29% over 60 d incubation period, highlighting the importance of extended incubation period in biodegradation studies (Fig. 5). These findings aligns with the previous study by (Olivito, Jagdale and Oza 2023a, b) where, in a soil burial study, 35% of weight loss was observed after initial one month of incubation and increased to 45% after extending the incubation period . Similarly, (Kim et al. 2022b) achieved up to 20% degradation of PU foam within 14 d of incubation with a bacterial strain HY-72. Another study by (Álvarez-Barragán et al. 2016) reported 65.3% of weight loss of PU foam by *Cladosporium tenuissimum* following 21 d of incubation.

While gravimetric weight loss is commonly used to assess polymer degradation; however, it may also be influenced by factors such as biomass interference, including attachment and adsorption of fungal biomass onto the polymer surface, or loose bound polymer particles during processing. In this study, SDS-assisted sonication followed by repeated washing and ethanol treatment was employed to remove adherent biomass prior to final weight measurement. Control samples processed under identical conditions did not show noticeable weight loss or fragmentation, suggesting minimal contribution from experimental handling. Nevertheless, some loss of fine polymer particles during processing cannot be entirely excluded. Therefore, the observed weight reduction was further examined alongside SEM, FTIR, XRD, and GC-MS analyses, which collectively suggests structural and chemical changes are consistent with PU biodegradation.

Although Impranil and PU foam differ both in their physical and chemical forms, both share soft-segment ester functionalities, that is highly prone to enzymatic hydrolysis in a similar manner, while variations in activity can be attributed to differences in the substrate accessibility as Impranil being a colloidal liquid dispersion allows easier enzyme access to the polymer chain, compared to PU foam which is solid and structurally more complex limiting enzymatic access, which eventually results in lower degradation reflected by 31.29% weight loss, whereas Impranil a liquid dispersion allows greater enzymatic accessibility and higher apparent mineralization. As these substrates were evaluated using different measurement approaches, the resulting percentages describe distinct biodegradation aspects and are not directly comparable.

The biodegradation efficiency of *Fusarium parceramosum* observed in the present study was compared with previously reported PU degrading microorganism. Several fungal species have demonstrated significant degradation potential; *Embaria clematidis* achieved 88.84% degradation of Impranil within 14 days (Khruengsai et al. 2022), while *Cladosporium halotolerans* exhibited 80% degradation within 3 days (Zhang et al. 2022). Similarly *Cladosporium tenuissimum* has been reported to degrade PU foam by 65.3% over 21 d (Álvarez-Barragán et al. 2016), and *Cladosporium* sp.* P7* exhibited significant degradation of PU foam waste, achieving a weight loss of 15.3%, under sole carbon source conditions and 83.83% in the presence of co-carbon sources after 15 d of incubation (Liu et al. 2023). *Xanthomonas* sp.* HY-71* exhibited degradation of PU foam, with PS PU and PE PU foams showing weight losses of 23.95% and 10. 95%, respectively, after 2 weeks of incubation (Kim et al. 2022a). Microbial consortia have also demonstrated enhanced degradation efficiencies, achieving up to 71% degradation under soil conditions (Gunawan et al. 2020). In addition to fungal strains, several bacterial strains have also been reported to exhibit high PU degradation efficiency under optimized conditions. For example, *Bacillus* sp.* YXP1* achieved complete mineralization of Impranil within 7 d and up to 42.1% degradation of PU foam after 30 d (Ji et al. 2024). Similarly, *Pseudomonas putida* demonstrated 92% degradation of Impranil within 4 d under optimized conditions (25 °C, pH 8.4) (Peng et al. 2014). Furthermore, *Bacillus velezensis* MB01B showed rapid degradation efficiency, achieving 91.4% mineralization of Impranil within 24 h (Zeng et al. 2024). PU biodegradation has also been reported in black soldier fly larvae (*Hermetia illucens*), which were able to survive on PU foam over 16 d, resulting in visible surface erosion. FTIR analysis revealed hydrolysis of ester, ether, and urethane bonds, changes in gut microbial community, along with isolation of PU-degrading bacteria ( *Delftia* sp.* A2*), highlights the efficiency of microbial degradation process (Wang et al. 2026). In comparison, *Fusarium parceramosum* demonstrated 31.29% degradation of PU foam over 60 d, and 92% degradation of (1%) Impranil within 192 h, under unoptimized conditions. These findings provide a strong preliminary evidence of biodegradation by *Fusarium parceramosum*, highlighting the scope for future optimization studies for efficient biodegradation of PU. Additionally, assessing the impact of environmental conditions – including pH, temperature, and substrate concentration- may lead to improved degradation outcomes and a better understanding of the process involved. To further understand the degradation behaviour, kinetic modelling was conducted after evaluating the biodegradation of Impranil and the gravimetric weight loss of PU foam over time. The data set where then fitted to zero-order, first-order, Langmuir, and Freundlich models, (Figs. 6 and 7), (Table 1).

For PU foam, the degradation trend observed in the gravimetric analysis, characterized by a gradual reduction in weight over time, was best described by the first-order kinetic model (R^2^ = 0.9988), with the zero-order model also showing a comparable fit (R^2^ = 0.9984). The predominance of the first-order kinetics suggests that the degradation rate is dependent on the remaining polymer content, which aligns with the experimental observation of a decreasing degradation rate as time progresses. This indicates that as the accessible polymer fraction is reduced, the degradation process slows down, likely due to limited surface availability. This behaviour reveals that PU foam degradation is primarily governed by enzyme-mediated hydrolysis occurring at accessible surface sites, where microbial enzymes act on exposed functional groups. As degradation progresses, reduced surface area and limited penetration of enzymes into the compact polymer matrix restrict further degradation. In contrast, the poor fit of Langmuir (R^2^ = 0.3737) and Freundlich (R^2^ = 0.6774) models suggests that adsorption-driven heterogeneity effects are minimal in the case of PU foam (Fig. 6). This is consistent with its rigid and compact structure, where degradation is more likely govern by bulk polymer breakdown rather than surface mediated interactions. These observations further suggests that substrate accessibility and diffusion of enzymes into the polymeric matrix act as a rate limiting steps in PU foam degradation. For Impranil, the biodegradation profile, expressed as percentage degradation over time, showed a relatively steady increase, which was best fitted by the zero-order model (R^2^ = 0.9839), closely followed by the first-order model (R^2^ = 0.9827). The zero-order kinetic behaviour indicates that the degradation rate remains nearly constant throughout the process, independent of substrate concentration. This can be attributed to the dispersed nature of Impranil in the medium, which ensures continuous availability of the substrate to microbial enzymes, thereby maintaining a uniform degradation rate. This suggests that microbial degradation of Impranil follows a substrate utilization pattern where enzymes have continuous and uniform access to the polymer, allowing sustained enzymatic activity without significant limitation by substrate availability. Additionally, the comparatively good fit of Langmuir (R^2^ = 0.8964), and Freundlich (R^2^ = 0.8755) models for Impranil suggests the involvement of surface interactions and heterogenous degradation mechanisms (Fig. 7). The higher accessibility and increased surface area of Impranil particles likely enhance enzyme–substrate interactions, supporting adsorption-related kinetic behaviour alongside bulk degradation. However, observations at higher Impranil concentration indicate a slight reduction in degradation efficiency, suggesting that enzyme activity or enzyme substrate interactions may become limiting under elevated substrate conditions. Overall, the kinetic modelling results are in good agreement with the experimental observations, highlighting distinct degradation patterns for the two PU forms. While PU foam degradation is governed by concentration-dependent kinetics due to its solid and recalcitrant nature, Impranil exhibits near constant-rate degradation owing to its higher dispersion and accessibility. These results collectively indicate distinct degradation mechanisms, where PU foam undergoes surface-limited enzymatic degradation constrained by accessibility and diffusion, whereas Impranil degradation proceeds under conditions of continuous substrate availability with enzyme-driven kinetics. In addition to the differences in physical form, PU foam and Impranil also differ in their chemical composition and structural organization, which significantly influence their degradation behaviour. PU foam is typically a crosslinked rigid polymer with limited accessibility to degradable bonds, whereas Impranil is a polyester-based PU dispersion containing more hydrolysable ester linkages. These structural and chemical differences make Impranil more susceptible to enzymatic attack compared to PU foam. Hence, the rate-limiting factors differ between the two systems, with substrate accessibility and diffusion constraints dominating in PU foam, while enzymatic activity and enzyme substrate interactions play a more significant role in Impranil degradation. Therefore, while kinetic comparisons provide useful insights, the degradation behaviours of these substrates are inherently influenced by both their physiochemical properties and chemical nature, and should not be interpreted as directly equivalent systems. Beyond substrate-specific differences, kinetic modelling approaches have been widely utilized in environmental systems to understand degradation and removal mechanisms. A relevant study by (Ayawei et al. 2015) demonstrated that Congo red removal using Ni/Al-CO_3_ layered double hydroxide followed zero-order (R^2^ = 1) and first-order kinetics (R^2^ = 0.9998), enabling identification of rate controlling steps and adsorption behaviour. Another similar study by (Edet and Ifelebuegu 2020) was on phosphate removal using recycled brick adsorbents applied kinetic modelling to understand adsorption mechanisms and rate-limiting steps. The process followed pseudo-second-order kinetics, indicating chemisorption dominance, with a Langmuir adsorption capacity of 5.35 mg/g. This further supports the use of kinetic models to interpret complex degradation behaviours. A related study on bio-based PU foams for oil–water separation employed kinetic modelling to understand sorption behaviour and rate mechanism. The process followed pseudo-second-order kinetics, with Langmuir and Redlich-Peterson isotherms providing the best fit, highlighting the relevance of kinetic modelling in interpreting PU-based systems (Olivito et al. 2023a, b). Similar, kinetic modelling of PU thermal degradation using model-free approaches (Kissinger, Friedman, Starink, and AIC) enabled evaluation of activation energy without assuming a reaction mechanism. The study revealed multi-step degradation behaviour, highlighting that kinetic modelling is crucial for capturing the complexity and stage-wise nature of polymer degradation processes (Zhao et al. 2024). Although kinetic modelling provides valuable insights into degradation mechanisms, its application in PU biodegradation studies remains relatively limited. In this context, the present study applies this approach to provide clearer insights into PU degradation, offering a more systematic and informative perspective compared to existing literature.

To complement kinetic modelling results, enzyme assays were conducted to assess *Fusarium parceramosum’s* hydrolytic potential on different PU substrates. *Fusarium parceramosum* has exhibited significant enzymatic activity (urease and aliphatic carbamate-hydrolyzing) during PU biodegradation (Fig. 8). PU is a heterogeneous polymer comprising urethane, ester, ether and urea functional groups, characterized by the presence of both C–N and C–O linkages within its backbone. The presence of carbonyl groups in these functional moieties facilitates nucleophilic attack on the carbonyl carbon, resulting in the cleavage of adjacent C–N or C–O bonds. In particular, C–N linkages present in urethane, amide, and urea groups are susceptible to enzymatic hydrolysis by hydrolases such as urethanases (aliphatic carbamate hydrolase), amidases, and ureases (EC 3.5.1). Among these, urethane (carbamate) bonds represent the primary structural linkage in PU and contain both C–N and C–O bonds, making them susceptible to enzymatic attack via urethanolytic and esterolytic pathways. Urethanolytic enzymes specifically target the C–N bond of urethane linkages, catalysing their hydrolysis into an amine and an unstable alkyl carbonate intermediate, which subsequently decomposes into an alcohol and carbon dioxide (Raczyńska et al. 2024). While the susceptibility of PU to enzymatic hydrolysis is governed by its chemical structure and bond composition, the actual degradation process in natural systems is initiated and driven by microbial colonization and enzyme secretion. Microbial colonization represents the initial step in PU biodegradation, wherein microorganisms such as bacteria and fungi adhere to the polymeric surface and form biofilms. These biofilms enhance surface attachment and create a localized microenvironment that facilities efficient enzymatic degradation. Following colonization, microbes secrete extracellular enzymes, including urethanases, esterases, and lipases, which act synergistically to hydrolyse urethane and ester linkages, leading to polymer chain scission and the formation of low-molecular-weight compounds that can be further metabolized. Urethanases identified from metagenomic libraries have demonstrated the ability to degrade low-molecular-weight dicarbamate intermediates generated during the chemical glycolysis of poly-ether PU foams, highlighting their potential in enzymatic recycling strategies. Furthermore, biological degradation of PU offers an environmentally sustainable approach to plastic waste management, as microorganisms such as bacteria and fungi produce enzymes including esterases, lipases, and ureases that contribute to the breakdown of polymer chains, thereby enhancing the recyclability and safe disposal of PU (Antaliya et al. 2025; Srikanth et al., 2022). In a recent study, PU degradation by an extracellular urethanase-producing bacterial isolate *Moraxella catarrhalis* was demonstrated using Berthelot reaction to quantify enzyme activity. Urethanase activity increased progressively during incubation, reaching a maximum of 1.639 mM min^− 1^ mg^− 1^ by 25 d, indicating active enzymatic involvement in polymer degradation. The hydrolysis of urethane groups resulted in the formation of equimolar amounts of amine, alcohol, and CO_2_, confirming direct cleavage of urethane linkages . Notably, when PU served as the sole carbon source, elevated activities of lipase, esterase, and urethanase were observed, with urethanase showing peak activity at later stages, highlighting its major role in sustained degradation (Maheswaran et al. 2024). Fungal biodegradation of PU is mediated by substrate-inducible enzymes, including esterases, proteases, and amidases, which hydrolyse urethane, ester and amide linkages within the polymer. Ureases contribute specifically by acting on urea-containing segments, thereby supporting degradation of certain PU variants. Additionally, a urethane hydrolase from the soil fungus *Exophiala jeanselmei* has been reported to directly cleave urethane groups, further confirming the role of enzymatic hydrolysis in fungal PU degradation (Maestri et al. 2023). Bacterial urethanases are known to efficiently break down urethane bonds due to their high catalytic activity and well-preserved active sites, making them useful for PU degradation and recycling. They are capable of hydrolysing carbamate groups effectively and continue to function under wide range of pH and temperature conditions, which supports their role as stable biocatalysts for PU biodegradation (Rotilio et al., 2026). Additionally, urease also facilitates hydrolysis of urea linkages into smaller molecules that can be further utilized by microorganisms. In some fungal systems, such as *Cladosporium pseudocladosporioides*, both esterase and urease activities have been associated with degradation processes, where disubstituted urea linkages are enzymatically converted into amines and CO_2_, further supporting polymeric breakdown (Skleničková et al. 2022). Microorganisms isolated from PU foam waste were evaluated for their enzymatic activity and their ability to grow on PU as a sole carbon source. It was observed that a majority of the strains (approximately 80%) produced urease, making it one of the most frequently detected enzymes. Several of these urease-producing microorganisms (*Acremonium strictum*, *Aspergillus fumigatus*, *Epicoccum nigrum, Penicillium* spp., *Phoma* spp., *Trichoderma* spp.), were also able to grow on PU-containing media, indicating a potential role in degradation. During polymer formation, reactions involving isocyanate groups and water lead to the generation of urea bonds. Urease act on these urea-containing segments, contributing to their breakdown into smaller compounds that can be further utilized by microorganisms. The high occurrence of urease-producing strains, together with their growth on PU as the sole carbon source suggests that urease play a major role in PU degradation process by facilitating the breakdown of urea linkages, thereby supporting subsequent microbial metabolism (Kemona and Piotrowska 2016). Collectively these findings demonstrates that carbamate-hydrolyzing enzymes directly mediate the cleavage of urethane linkages in PU, while urease contributes through hydrolysis of urea-containing segments, collectively providing mechanistic linkage between enzyme activity and PU breakdown. This highlights the distinct roles of enzymes in PU degradation. Nevertheless, due to steric hindrance and the complex three-dimensional structure of PU, direct enzymatic accessibility to urethane linkages within intact polymer chains may be limited, often requiring prior depolymerization or synergistic enzymatic action. Collectively, these findings indicate that urease contributes to PU degradation in a supportive role by acting on urea-containing functionalities, whereas urethanases play a more direct and mechanistically significant role in the breakdown of urethane linkages within the polymer structure. Since these functional group are present in both the PU types, the same enzymatic system appears to be responsible for degradation of both the materials. Difference observed in degradation efficiency are therefore likely related to structural variations in polymer composition and accessibility rather than the involvement of distinct enzyme systems. In the present study, over 60 d incubation period with *Fusarium parceramosum*, the activities of both the enzymes followed a gradual decline over time. This trend may be attributed to substrate depletion, which reduces enzyme induction as the available polymer fraction decreases, as well as feedback inhibition caused by accumulation of degradation intermediates which is commonly seen in biodegradation studies. Additionally, environmental stress factors, such as pH variations or accumulation of potentially inhibitory byproducts, may further contribute to the reduction in enzyme activity. This decline in enzyme activity corresponds with the observed decrease in degradation rate at later stages, indicating that enzyme activity is closely linked to the biodegradation process and may become a limiting factor over time, particularly in PU foam where substrate accessibility is restricted. Complementing this enzyme assay using Impranil showed higher enzyme activity during initial stage can be attributed to its dispersed nature, which allows better enzyme accessibility and substrate utilization. However, at higher substrate concentrations, a reduction in enzymatic activity was observed, suggesting possible substrate inhibition or limitations in enzyme-substrate interactions, indicating that hydrolysis is more efficient under moderate substrate conditions. A similar declining trend in enzymatic activities over incubation time has been reported by (Khruengsai et al. 2022) in their study.

In addition, PU biodegradation within the genus *Fusarium* has been associated with diverse enzymatic systems. *Fusarium Venettenii* and *Fusarium solani* have been reported to degrade PU materials through diverse enzymatic systems including esterase, lipase, protease, peroxidase, and laccase activities (Ren 2021; Okal et al. 2025). In contrast, the present study identified urease and aliphatic carbamate hydrolase as the predominant enzymes associated with PU biodegradation in *Fusarium parceramosum*, while esterase and lipase activities were negligible under tested conditions. This indicates that although PU biodegradation may be a broader trait within the genus *Fusarium*, the specific enzymatic activity involved varies among species. Moreover urease and aliphatic carbamate-hydrolyzing activities have been documented in bacterial PU-degrading systems (Oceguera-Cervantes et al. 2007; Fuentes-Jaime et al. 2022), suggesting that cleavage of carbamate and urea linkages represents a common biochemical strategy across different microbial taxa. However, it was not determined whether these activities (urease and aliphatic carbamate-hydrolyzing activities) originate from a single multifunctional enzyme or from multiple enzyme isoforms. In addition, the genetic basis underlying these enzymatic activities were not investigated, in the present study. Future studies involving gene identification and transcriptomic analysis would provide deeper insights into the molecular mechanisms governing these processes.

Furthermore, while most studies relay on agar-based assay, spectrophotometric evaluation employed in the present study enabled precise quantitative assessment over time.

Complementing the gravimetric findings, kinetic modelling and enzyme analysis. SEM provided structural evidence of PU foam biodegradation by *Fusarium parceramosum*. Progressive surface cracking, tearing, erosion, and void formation reflect sustained enzymatic and structural disruption of PU foam matrix exclusively in *Fusarium parceramosum* incubated samples and were absent in abiotic control reflecting highlighting the degradation efficiency of *Fusarium parceramosum* over extended incubation period. These morphological changes, together with the detected extracellular urease and aliphatic carbamate activities, suggest that enzymatic hydrolysis may have contributed in weaking of the polymer structure. In addition, such surface alterations may also be influenced by mechanical disruption caused by fungal hyphal penetration and growth, which can exert physical stress on the polymer matrix, leading to surface cracking, and structural weakening of the polymeric matrix which enhance polymer accessibility and contribute to overall material deterioration. (Zhu et al. 2025), reported 21.2% of weight loss of PU foam in their study, accompanied by SEM evidence, where prominent surface cavities were observed following bacterial laccase treatment. Similarly, (Khan et al. 2020) observed progressive surface disruption by *Aspergillus flavus* G10, including holes, pits and cracks following 28 d of incubation. Also, (Álvarez-Barragán et al. 2016) in their study observed large hyphal invasion, visible surface cracking and thinning of PU foam surface by *Cladosporium tenuissimum* following 21 d of incubation. In contrast, the present study provides a detailed time-course analysis of *Fusarium parceramosum*-mediated degradation (Fig. 9) of PU foam under unoptimized conditions, complementing existing literature.

In line with the structural changes observed via SEM, FTIR analysis revealed chemical modification in PU foam incubated with *Fusarium parceramosum*. The control sample exhibited characteristic peak corresponding to PU matrix, indicating intact aliphatic carbamate linkage and unaltered functional groups. In contrast, the treated sample demonstrated a slight shift, and reduction in the intensity of these peaks (Fig. 10), particularly in the N–H stretching region, which is associated with aliphatic carbamate and hydrogen bonds essential for polymer stability. Changes were also observed in the aliphatic C–H stretching vibrations, indicative of the hydrocarbon chains within the soft segments. Furthermore, the ester (C=O) peak, which confirms the presence of ester bond in the polymeric backbone, and the aliphatic ether (C–O) peak, characteristic of polyol chains in the soft segment, both exhibited reduced intensities, indicating alterations in the polymeric framework. A minor absorption at 2210 cm^− 1^ in treated sample indicates nitrile or isocyanate derivatives produced during polymer breakdown (Table. 2). These observations indicate the initial hydrolysis of aliphatic carbamate bonds along with alteration in hydrogen bonding, suggesting that depolymerization of the treated PU was initiated predominantly within the soft segments. These chemical alterations align with previous reports of fungal mediated PU degradation by (Amaral et al. 2012; Maestre-López et al. 2024). Although the spectral shifts were minor, they reflect underlying chemical changes such as oxidation, hydrolysis and chain scission within the polymer structure, which has been reported in polymer degradation studies (Prakash Bhuyar et al., 2019).

After observing the chemical modifications in the polymeric backbone, the control and the *Fusarium parceramosum*-treated PU foam were analysed for their changes in crystalline structure. The treated sample exhibited a decrease in peak intensity and overall crystallinity (from 36.6 to 13.4%) over 60 d incubation period with *Fusarium parceramosum* (Fig. 11), indicating the disruption of the ordered domains in the polymeric matrix while still retaining partial crystallinity (Table. 3). The observed decrease in crystallinity and peak intensity suggests that degradation was preferentially initiated in the amorphous region of the polymer, which are more accessible to microbial attack. PU typically exhibits a semi-crystalline structure, where amorphous regions are less ordered and loosely packed, whereas crystalline regions are highly ordered and densely packed, making them comparatively more resistant to degradation. Disruption of these less ordered domains alters the overall structural organization of the polymer matrix, thereby affecting crystalline regions and resulting in a reduction in crystallinity. These findings align with the study of (Pfohl et al. 2022), where degradation of polylactic acid ( PLA) and polyglycolic acid ( PGA) with their copolymers was studied in simulated marine environment. It was observed that decrease in peak intensity occurred during the degradation process, but peak position remained unchanged, indicating the retention of the fundamental crystal lattice. This interpretation proves that biodegradation sometimes leads to partial disruption of crystallinity without total amorphization, which aligns with the results obtained in the present study. Biodegradation can also lead to increase in overall crystallinity when selective degradation of amorphous region occurs, which was observed in the findings of (Chen et al. 2024). Based on the observed alterations and consistent literature findings, it appears that changes in the polymer’s crystallinity is largely determined by the nature of the polymer and the interaction between the polymer and microbial enzymes. In the present study *Fusarium parceramosum* showed effective degradation of the polymer’s matrix predominantly by depolymerization of the soft segment domains , highlighting its biodegradation potential.

**Fig. 14:**
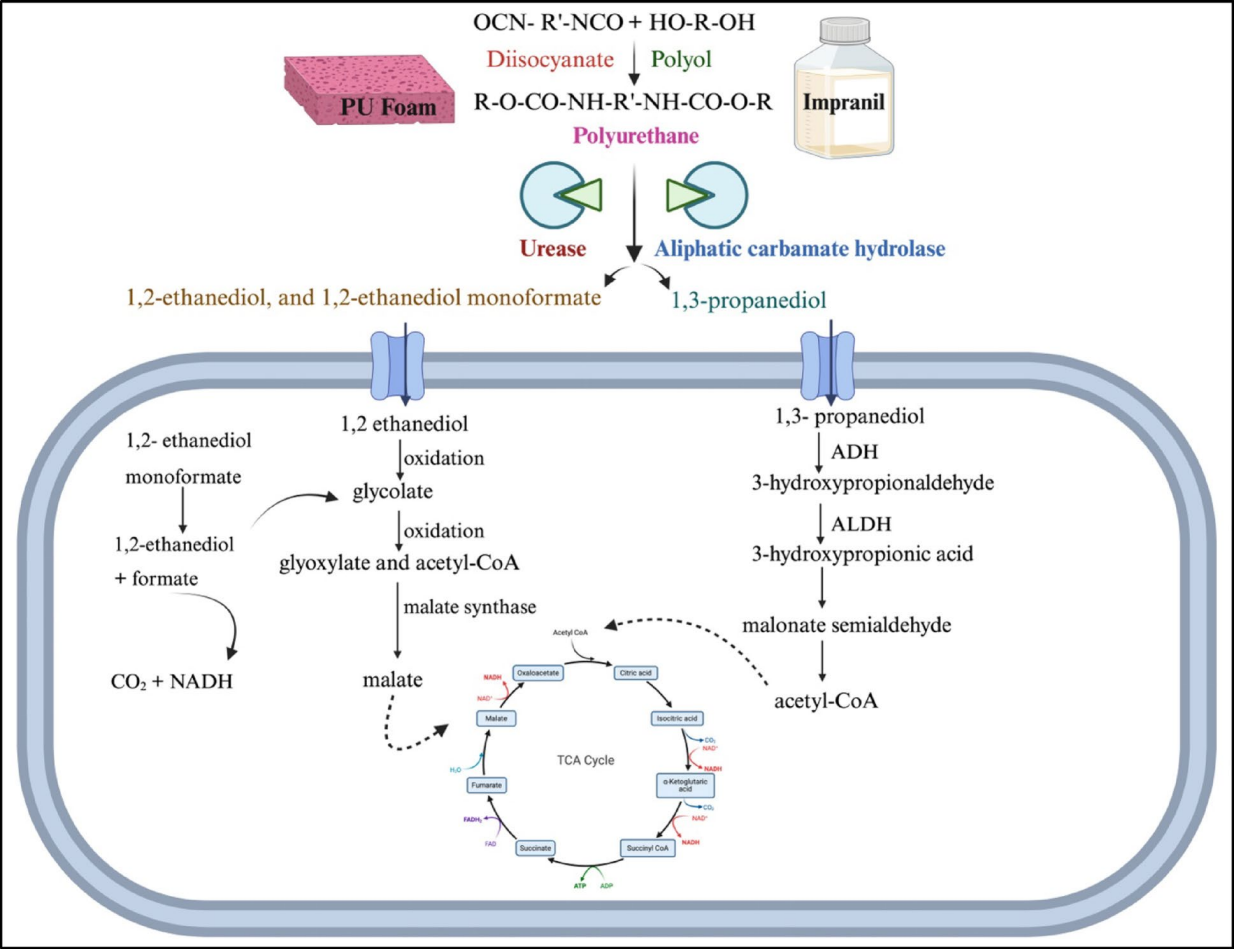
Putative PU degradation pathway based on GC–MS-identified intermediates. 1,2-ethanediol and monoformate are derived from PU foam, while 1,3- propanediol originates from Impranil

Following XRD, the samples were further analysed to characterise the degradation intermediates using GC–MS, where presence of 1,2 ethanediol, and 1, 2 ethanediol monoformate was exclusive to the *Fusarium parceramosum*-treated foam sample (Table 4). The presence of these intermediates indicates the hydrolytic and oxidative mechanism responsible for breakdown of the soft segment (Fig. 12). These results align with the previous reports of (Sandten et al. 2024), where mono- and di-formates of glycol were synthesized under in-situ conditions and were used as calibration standards to detect glycol ester intermediates by GC–MS. Following the detection of PU degradation intermediates for the treated foam sample, the analysis of degradation was done for the *Fusarium parceramosum*-treated Impranil sample, where 1,3-Propanediol was detected solely in the treated sample (Table 4), confirming hydrolytic breakdown of polyester segments (Fig. 13). A similar finding was reported by (Zhang et al. 2022), where 3-dipropylamino-1,2-propanediol was detected as a degradation intermediate in *Cladosporium halotolerans* 6UPA1-treated Impranil sample. Additionally, based on the intermediates detected in this study, a putative metabolic pathway is proposed, indicating that these intermediates might be potentially linked to central metabolic pathway (Fig. 14). The detection of 1,2- ethanediol and 1,2-ethanediol monoformate in *Fusarium parceramosum*-treated PU foam sample indicating that soft-segment hydrolysis generates low-molecular weight diols that are metabolically accessible. 1,2 -ethanediol can undergo oxidative conversion to glycolate and subsequently to glyoxylate, which may be assimilated via the glyoxylate shunt through condensation with acetyl-CoA to form malate, a tricarboxylic cycle (TCA) intermediate. The monoformate derivative further gets hydrolyzed to release ethylene glycol and formate; while ethylene glycol follows the same assimilative route, formate can be oxidized to CO_2_, contributing reducing equivalents for cellular energy metabolism (Balola et al. 2024). In addition to 1,2- ethanediol and 1,2-ethanediol monoformate, 1,3-propanediol was also detected as a degradation intermediate in *Fusarium parceramosum*-treated Impranil sample supporting the hydrolysis of soft segments. 1,3-propanediol can undergo oxidation to 3-hydroxypropionaldehyde by alcohol dehydrogenases and further to 3-hydroxypropionic acid by aldehyde dehydrogenases. 3-hydroxypropionic acid can be metabolized via malonate semialdehyde to acetyl -CoA, which represents the entry point into TCA cycle and central metabolism (Stevens et al. 2011). Consistent with this, a study on *Cladosporium* sp.* P7* proposed that degradation intermediates may undergo further biochemical transformations into metabolites associated with central metabolic processes (Liu et al. 2023). Similarly, PET degradation by *Paenibacillus naphthalenovorans* has been reported, where PET microplastics were gradually degraded over 35 d incubation period. Metabolite analysis revealed the presence of several intermediate compounds during this process, by integrating these findings with genomic data, a probable degradation pathway was proposed, illustrating the conversions of large polymeric molecules into smaller molecules. Such insights contribute to a clearer understanding of the mechanisms underlying microbial plastic degradation (Choonut et al. 2026). Together, these observations suggests that the detected compounds function as metabolically accessible intermediate rather than end products, thereby supporting fungal growth and energy metabolism.

Although degradation products were detected, the possibility of generating undesirable secondary metabolites during fungal PU metabolism cannot be completely ruled out. However, GC–MS analysis performed in the present study did not reveal the presence of any toxic or unexpected secondary metabolites among the detected compounds. Overall, *Fusarium parceramosum* mediated degradation causes progressive weight loss and structural and chemical modifications. This highlights the metabolic versatility and potential of *Fusarium parceramosum* for efficient PU biodegradation, predominantly via soft segment depolymerization.

## Conclusion

The present work documents the first evidence of PU degradation by a soil-borne fungus *Fusarium parceramosum*, with two chemically different PU substrates Impranil (a PU dispersion) and PU foam, under unoptimized conditions. Preliminary evidence of degradation was confirmed by the formation of halo zone (within 5 d) on MSM-Impranil agar plates. Quantitative analysis of Impranil biodegradation showed substrate inhibition at higher Impranil concentrations. For PU foam 31.29% of gravimetric weight loss was reported as a response of biodegradation after 60 d incubation. Although PU foam exhibited a maximum weight loss of 31.29% over 60 d, it is a highly recalcitrant polymer that persists in the environment for extended periods. Therefore, such partial degradation under unoptimized laboratory conditions demonstrates biodegradation potential. Kinetic modelling of degradation data using zero-order, first-order, Langmuir, and Freundlich models revealed that zero-and first-order models provided the best indicating that degradation predominantly followed concentration-dependent kinetics rather than adsorption-controlled mechanism.

Consistent with this, the gradual decline in urease and aliphatic carbamate-hydrolyzing activities over time reflects reduced substrate accessibility within the solid polymer matrix. Degradation efficiency could be further enhanced through optimization of culture conditions, including adjustment of pH, temperature, and agitation speed, in addition of appropriate carbon and nitrogen sources, application of substrate pretreatment strategies such as UV exposure, may induce surface oxidation and increase polymer susceptibility to enzymatic attack. In addition to these findings SEM revealed characteristic surface alterations. FTIR showed hydrolysis of aliphatic carbamate bond and emergence of new peak indicating the formation of derivatives during the biodegradation process. XRD indicated a crystallinity drop from 36.6% to 13.4% and GC–MS detected intermediates such as 1,2-ethanediol, 1,2-ethanediolmonoformate, and 1,3-propanediol, supporting hydrolytic and oxidative degradation. Based on these intermediates, a putative pathway is proposed, suggesting that these compounds may undergo sequential transformations, potentially forming products that may participate in microbial metabolic processes. Overall, these results confirm *Fusarium parceramosum*’s sustained enzymatic efficiency and strong degradation potential for dual PU substrate degradation through structural, chemical and enzymatic mechanisms, supporting sustainable waste management and contributing to responsible consumption and environmental protection. The ability to act on diverse substrates indicates its suitability for PU waste management, including applications in controlled bioreactor environments, composting systems, and in combination with microbial consortia to enhance degradation of diverse PU waste. However, further studies are required to evaluate its performance under field conditions and optimize process parameters for large-scale applications. Overall, this study aligns with SDG 12 (Responsible consumption and production) by promoting eco-friendly plastic degradation, SDG 13 (Climate action) by reducing pollution-related emissions, and SDG 14 (Life below Water) and SDG 15 (Life on Land) by mitigating PU contamination in aquatic and terrestrial ecosystems.

## Data Availability

All the required data are provided within the manuscript.
